# Supplementary material for: Green synthesis of stable hybrid biocatalyst using a hydrogen-bonded, π-π-stacking supramolecular assembly for electrochemical immunosensor
Source: Nat Commun. 2023 Jun 20;14:3644. doi: 10.1038/s41467-023-39364-x (PMC10282088; doi:10.1038/s41467-023-39364-x)
Supplement: Supplementary file 1 — Supplementary Information [file 41467_2023_39364_MOESM1_ESM.pdf]

Supplementary Information

Green synthesis of stable hybrid biocatalyst using a hydrogen-bonded, π-π-stacking supramolecular assembly for electrochemical immunosensor

Wei Huang,[1,2] Haitao Yuan,[3] Huangsheng Yang,[2] Xiaomin Ma,[4] Shuyao Huang,[5] Hongjie Zhang,[2] Siming Huang,[6] Guosheng Chen,[2]* and Gangfeng Ouyang[1]*

[1]School of Chemical Engineering and Technology, Sun Yat-sen University, Zhuhai 519082, China

[2]MOE Key Laboratory of Bioinorganic and Synthetic Chemistry, School of Chemistry, Sun Yat-sen University, Guangzhou 510275, China

[3]Department of Geriatric Medicine, Shenzhen People's Hospital (The Second Clinical Medical College, Jinan University), Shenzhen 518020, China

[4]Cryo-EM Center, Southern University of Science and Technology, Shenzhen, 518055, China

[5]Instrumental Analysis and Research Center, Sun Yat-sen University, Guangzhou 510275, China

[6]Guangzhou Municipal and Guangdong Provincial Key Laboratory of Molecular Target & Clinical Pharmacology, the NMPA and State Key Laboratory of Respiratory Disease, School of Pharmaceutical Sciences and the Fifth Affiliated Hospital, Guangzhou Medical University, Guangzhou, 511436, China

*Corresponding authors, E-mail: chengsh39@mail.sysu.edu.cn (G. Chen); cesoygf@mail.sysu.edu.cn (G. Ouyang)

1. Supplementary Methods

1.1. Reagents and materials

Enzymes including horseradish peroxidase (HRP, from horseradish, >300 U/mg), glucose oxidase (GOx, from Aspergillus niger, >180 U/mg), cytochrome c (Cyt c, from Equus caballus heart, >95%), trypsin, urease, *candida rugosa* Lipase and other reagents including cholesterol (>95%), glucose (AR), chloroplatinic acid hydrate ($H_2PtCl_6 \cdot xH_2O$, 99.95%), sodium borohydride ($NaBH_4$, 98%), poly-(diallyldimethylammonium chloride) (PDDA, 20wt%) and 3,3',5,5'-tetramethylbenzidine (TMB, 99%) were obtained from Aladdin Chemistry Co., Ltd. (Shanghai, China). Phosphotungstic acid negative stain solution (5%) was purchased from Solarbio Technology Co., Ltd. Bovine serum albumin (BSA, >98%) and myoglobin (MB) were purchased from Sigma-Aldrich (Shanghai, China). 1,3,6,8-tetracarboxy pyrene (H_4TCPy, 95%) was purchased from J&K Scientific (Beijing, China). Hydrogen tetrachloroaurate-(III) hydrate ($HAuCl_4 \cdot 4H_2O$, Au > 47.5%) was purchased from Strem Chemicals. Hydroquinone (HQ, AR) were bought from Sangon Biotechnology Co., Ltd. Rhodamine B (RhB), p-nitrophenyl butyrate (NPB, >98%), p-nitrophenyl palmitate (p-NPP, >98%) and p-nitrophenol (p-NP, >99%) were purchased from Macklin Biochemical Technology Co., Ltd. (Shanghai, China). Dimethyl formamide (DMF, 99.5%) was purchased from Guangzhou Chemical Reagent Factory (Guangzhou, China). Hydrogen peroxide (H_2O_2, 30%), $K_4[Fe(CN)_6]$ (98%) and $K_3[Fe(CN)_6]$ (>99.5%) were obtained from Beijing Chemical Reagent Co. (Beijing, China). Human Mucin 1 (MUC1) and MUC1 antibody (Ab1 and Ab2) were purchased from Boosen Biotechnology Co. Ltd (Beijing, China).

All chemicals and reagents were purchased from commercial sources and used without further purification. All serum sample studies were approved by the Medical ethics committee of the Third Affiliated Hospital of Guangzhou Medical University (Project number: Medical ethics joint trial [2021] NO. 001). Informed written consent from all participants was obtained prior to the research. The experiments were carried out following the guidelines of the National Institutes of Health of China for the care

and use of tissue samples.

PBS work buffer (0.1 M Na_2HPO_4, 0.1 M KH_2PO_4 and 0.1 M KCl, pH 7.4) was used as working buffer. In this work, all the solutions were prepared using ultrapure water, which derived from Milli-Q water purification system with an electric resistance of 18.2 MΩ.

1.2. Characterization

CHI 750E electrochemistry workstation (Shanghai Chenhua instrument, China) was used to operate differential pulse voltammetry (DPV) measurements. DPV measurements were performed in 0.1 M PBS solution (pH 7.4) with the potential from 0.2 V to -0.35 V, the step potential of 4 mV and the amplitude of 50 mV. Cyclic voltammetry (CV) and electrochemical impedance spectroscopy (EIS) experiments were recorded on a CHI 750E electrochemical workstation (Shanghai, China). A classic three-electrode system was adopted, the modified gold electrode (GE) (Φ = 3 mm) served as working electrode, a platinum wire served as a counter electrode and Hg/Hg_2Cl_2 as the reference electrode.

Powder X-ray diffraction (PXRD) patterns were collected (0.02°/step, 0.06 seconds/step) on a Bruker D8 Advance diffractometer (Cu Kα) at room temperature.

N_2 adsorption isotherms were collected with a JW-DX Surface Area Analyzer at -196 °C. All the samples were pre-treated under 100 °C for 12 h before measurements.

The ultraviolet-visible (UV-Vis) absorbance measurement was carried out on a 2800S spectrophotometer (SOPTOP, Shanghai), while the UV-Visible diffuse reflectance spectrum was performed with a 3600 spectrophotometer (Shimadzu, Japan).

Fourier transform infrared (FTIR) spectroscopy was performed with a Bruker EQUINOX 55 spectrometer (32 scans in the 4000-400 cm^{-1} spectral range).

Thermogravimetric analyses (TGA) were performed under N_2 atmosphere (20 mL min^{-1}) with temperature increasing at 10 °C min^{-1} using a TA-Q50 system. The samples were dried in vacuo at 100 °C for 12 h before TGA analysis.

Zeta potentials were measured on a Nano ZS 90 system.

The morphology images of the crystals were taken on a SU8010 ultra-high resolution

field emission scanning electron microscope (SEM, Hitachi, Japan). Transmission electron microscope (HRTEM) and high-angle annular dark-field scanning transmission electron microscopy (HAADF-STEM) images were taken on a JEM-2010HR microscope operating at 200 kV.

Circular dichroism (CD) spectra of enzymes were analyzed by a J1700 CD Spectrometer (JASCO, Japan) in the spectrum region 190-300 nm.

Matrix-assisted laser desorption ionization time-of-flight mass spectrometry (MADIL-TOF MS) analysis of chemical modified proteins was performed on an ultrafleXtreme MALDI-TOF/TOF-MS (Bruker Daltonics, Bremen, Germany).

Solid state nuclear magnetic resonance (ssNMR) spectra were recorded on Bruker AVANCE III 400MHz spectrometers.

Electron paramagnetic resonance (EPR) experiment was carried out on Bruker EMX plus 10/12 equipped with Oxford ESR910 Liquid Helium cryostat. The test temperature was set at 5 K.

Confocal laser scanning microscope (CLSM 880 NLO, Carl Zeiss, Göttingen, Germany) was used to determine the distribution of dye-labelled enzyme within HOF-100.

Cryo-electron microscopy (Cryo-EM) experiments were performed on a ThermoFisher Scientific Titan Krios G3i electron microscopes operated at 300 kV. The dispersive nanomaterials (in ethanol) were mounted to a carbon-coated TEM-grid and dried under vacuum. The specimen was then dropped into liquid nitrogen and transferred by a Cryo-transfer loader into the microscope. Cryo-EM images were collected by a K3 Summit direct electron detector equipped with a GIF Quantum energy filter (slit width 20 eV) in the counting mode (Bin 0.5). Data acquisition was performed using SerialEM 3.864 with a nominal magnification of 350,000 ×, corresponding to a physical pixel size of 0.34 Å. The dose rate was ~15 counts/pixel/second, and the exposure time in a frame was 0.023 s. Each micrograph stack contains 10 frames (the total exposure time was 0.23 s), and the total dose rate was ca. 30 e-/Å^2 per micrograph. The motion correction was performed using MotionCorr265 with 2×2 binning, and the non-dose-weighted sum of all frames from each movie was used for all image

processing steps. The lattice spacing of enzyme@HOF-100 unit cells were analyzed using DigitalMicrograph (Gatan) software.

1.3. Synthesis of standard HOF-100

The standard HOF-100 was prepared in organic solvents according to the reported method.[1] 20 mg H_4TCPy was dissolved in 1 mL of DMF under heating 120 °C for 30 min to get a clear solution. After cooling down room temperature, the solution was poured into 16 mL of acetone under stirring within 1 min. The suspension was stood at room temperature for 12 hours and isolated by centrifugation at 6080 g for 5 min. Finally, the product was collected by centrifugation, washed by acetone and then dried under vacuum at room temperature.

1.4. Synthesis of GOx-Pt nanoclusters (GOx-Pt NCs)

In a typical synthesis of GOx-Pt NCs, GOx (20 mg) was dispersed in 10 mL PBS (pH = 7.4). 0.5 mL H_2PtCl_6 (38.6 mM) in PBS was added in the reaction solution, and the mix was magnetically stirred at dark for 30 min. After that, 0.25 mL $NaBH_4$ (0.5 M) was added in the reaction solution to reduce H_2PtCl_6. 2 h later, the reaction solution was transferred into a dialysis bag (Biosharp, molecular weight cut off = 1000 Da), and then was dialyzed against deionized (DI) water for several times.[2]

1.5. Synthesis of GOx-Pt@HOF-100

The raw enzyme was replaced by GOx-Pt NCs to prepare the GOx-Pt@HOF-100 as described in green synthesis of HOF hybrid biocatalysts.

1.6. Enzyme loading measurement in proteins@HOF-100

The loading content of proteins@HOF-100 (HRP, BSA, Cyt c, GOx, MB, trypsin and urease) was measured by examining the concentration differences of proteins in the supernatants before and after encapsulation *via* a standard Bradford assay[3]. Typically, 20 μL of enzyme sample was added into a 96-well plate, followed by introducing 200 μL of Coomassie Brilliant Blue G-250 reagent. After 5 min incubation, the solution was

collected and detected by UV-Vis spectrophotometer. The concentration of the enzyme was proportional to the absorbance at 595 nm.

1.7. Enzyme loading measurement in Lipase@HOF-100

In fact, we tried to quantify the Lipase using standard Bradford protein assay, but we found that the standard Bradford assay was not workable for Lipase. On the contrary, using standard bicinchoninic acid (BCA) assay[4], the concentration of Lipase was in direct proportion to the UV-Vis absorbance at 562 nm. Therefore, we chose the standard BCA assay for Lipase quantification.

The loading content of Lipase@HOF-100 was measured by examining the concentration differences of enzyme in the supernatants before and after encapsulation *via* a standard BCA proteins assay. Typically, 20 μL of enzyme sample was added into a 96-well plate, followed by introducing 200 μL of BCA working solution. After incubating at 60 °C for 15 min, the solution was collected and detected by UV-Vis spectrophotometer. The concentration of the enzyme was proportional to the absorbance at 562 nm.

1.8. Fluorescence labeling of enzymes

The fluorescence labeling experiments were carried out based on the conjugation between the amino of lysine residue of enzymes and the thiocarbamide of fluorescence dyes. In brief, 10 mg enzyme was dispersed into 1 mL of carbonate buffer solution (pH=9.0, 0.5 M), followed by adding 1 mg rhodamine B (RhB) isothiocyanate. The mixed solution was then stirred for 12 h in dark condition. Finally, the RhB-labelled enzymes were obtained through ultrafiltration by a centrifugal filter device (molecular weight cut-off MWCO= 8 kDa) for several times to remove excess reaction reagents and salts.

1.9. CLSM experiment

For the CLSM experiment, the Lipase/HRP was labelled by RhB isothiocyanate (denoted as RhB-Lipase/RhB-HRP). The raw Lipase/HRP was replaced by RhB-

Lipase/RhB-HRP to prepare the Lipase@HOF-100/HRP@HOF-100 as described above.

1.10. Enzyme adsorption experiment

The enzyme adsorption experiment was carried out to demonstrate that the enzyme was encapsulation into rather than absorbed onto HOF-100. In briefly, 5 mg HRP/Lipase was added to 9 mL of dispersive standard HOF-100 aqueous solution, and then dispersed by ultrasonic treatment. The mixed solution was stirred at room temperature for 5 min and aged for another 15 min. The experimental parameters including the enzyme dosage, stirring and aging times were similar as those in the assembly process of enzymes@HOF-100. Finally, the free HRP/Lipase in the supernatant was collected by centrifugation, and quantified by Bradford/BCA proteins assay.

The HRP/Lipase adsorption contents were measured by examining the concentration differences of free HRP/Lipase in the supernatants before and after adsorption. The very limited adsorption capacity of HRP/Lipase by HOF-100 indicated that enzymes were unable to infiltrate into the relative narrow pore channel of HOF-100 or be surface-absorbed on HOF-100, which further confirmed that the enzymes were encapsulated during the growth of HOF-100.

1.11. Protein negative staining method

We prepared protein negative staining sample using drip staining method. First, 5-10 μL sample was dropped on a copper mesh to form liquid bead. After standing for 1min, a small piece of filter paper was used to suck out excess liquid from the edge of the copper mesh. Subsequently, 5-10 μL of phosphotungstic acid negative stain solution was further dropped on the copper mesh to form liquid beads. After standing for another 1 min, excess liquid at the edge of the copper mesh was sucked away from by a small piece of filter paper. After drying, the prepared sample was examined by HRTEM.

1.12. Activity of Cyt c

The enzymatic activities of Cyt c or Cyt c@HOF-100 were evaluated by tracing the catalytic product of oxTMB at 650 nm using a UV-Vis spectrophotometer based on Equation 1:

$$H_2O_2 + TMB \xrightarrow{Cyt\ c} H_2O + oxTMB \qquad (1)$$

The concentration of free Cyt c was set at 33.3 μg/mL, and the concentrations of Cyt c@HOF-100 used in each trial were also controlled to be 33.3 μg/mL according to the calculated enzyme loading content (Supplementary Table 2). Typically, 200 μL of free Cyt c /Cyt c@HOF-100 solution and 200 μL of prepared TMB solution were mixed. After that, a series concentration of H_2O_2 (200 μL) was added to activate the catalytic reaction.

The produced oxTMB could be monitored at 652 nm by a UV-Vis spectrophotometer using a time-scanning mode.

1.13. Activity of GOx.

The enzymatic activities of GOx and GOx@HOF-100 were evaluated based on the time-dependent H_2O_2 generation (Equation 2).

$$glucose + O_2 \xrightarrow{GOx} gluconic\ acid + H_2O_2 \qquad (2)$$

The concentration of free GOx was set at 15 μg/mL, and the concentrations of GOx@HOF-100 used in each trial were also controlled to be 15 μg/mL according to the calculated enzyme loading content (Supplementary Table 2). Typically, 200 μL of free GOx/GOx@HOF-100 solution, 200 μL of HRP solution (12.5 μg/mL) and 200 μL of prepared TMB solution were mixed. After that, a series concentration of glucose (200 μL) was added to activate the catalytic reaction.

The catalytic product of H_2O_2 could be evaluated based on the HRP enzymatic reaction using TMB as the substrate, in which H_2O_2 was stoichiometrically converted into oxTMB. The generated oxTMB could be monitored at 652 nm by a UV-Vis spectrophotometer using a time-scanning mode.

1.14. Measurement of the catalytic kinetic parameters

The catalytic kinetic parameters were measured according to the Michaelis-Menten equation (Equation 3)[5]:

$$V_0 = \frac{V_{\max}[S]}{K_m + [S]} \qquad (3)$$

Here, V_0 is the initial catalytic rate; $V_{\max}$ is the maximum rate conversion, which is obtained when the catalytic sites on the enzyme are saturated with substrate. [S] is the initial substrate concentration, and K_m is the Michaelis-Menten constant. The initial catalytic rates V_0 was determined by making the slope of the kinetic curve in the initial phase (t from 0 to 30 s), and the initial substrate concentration [S] was determined at t=0 s. The kinetic parameters K_m and $V_{\max}$ were fitted using a Michaelis–Menten equation based on the calculated V_0 and [S]. The analysis was performed with the GraphPad prism version 5.0.1 (GraphPad Software, Inc., San Diego, CA).

1.15. Synthesis of Au NPs

Au NPs was synthesized by the sodium citrate reduction method.[6] Firstly, 1 mL of 1 wt% $HAuCl_4$ aqueous was added into 100 mL of H_2O. After heating it to boil, 2.5 mL of 1 wt% $Na_3C_6H_5O_7$ was quickly injected. The resulting mixture was kept boiling for about 15 minutes. When the solution color changed from yellow into wine-red, the Au NPs were formed. Ultimately, the obtained Au NPs solution was stored at 4 ^{o}C in brown reagent bottle for later use.

1.16. Synthesis of Ab2/Au NPs/HRP@HOF-100

5 mg HRP@HOF-100 was dispersed in 5 mL of PDDA aqueous solution (1 wt%) with shaking overnight at room temperature to form positively charged HRP@HOF-100. Following that, 1 mL of negatively charged Au NPs solution was added into the as-prepared HRP@HOF-100, along with shaking for 5 h at room temperature to obtain the Au NPs/HRP@-HOF-100 nanocomposite via the electrostatic interaction. Subsequently, the product was collected by washing with ultrapure water. Afterward, 20 μL of Ab2 (0.5 mg/mL) was injected into the above-prepared Au NPs /HRP@HOF-

100 solution (1 mL, 1 mg/mL) and stirred for 12 h 4 °C to form the Ab2/Au NPs/HRP@HOF-100 biocomposite through the Au-NH_2 interaction. The resultant product was centrifuged and finally dispersed in 1 mL of 0.1 M PBS (pH 7.4) and stored at 4 °C for further use.

1.17. The calculation of detection limit

The calculation of detection limit was according to the previous report[7], which was described as follows. DPV measurements for blank samples were executed with three parallel experiments, which exhibited an average photocurrent intensity (I_B) of -3.289 μA with standard deviation (S_B) of 0.085. With signal-to-noise ratio value (k) of 3, the smallest detectable signal (I_L) could be calculated as

$$I_L = I_B + 3\ S_B = -3.035\ \mu A$$

Then the value of I_L=-3.035 was inserted into the ultralow concentration linear equation:

$$I = -7.107 \times lgc - 8.418$$

Thus, the detection limit (c_L) was calculated to be 0.18 pg/mL.

Supplementary Tables

Supplementary Table 1. The isoelectric points and molecule weights of the proteins

Proteins	Isoelectric point (PI)[a]	Molecule weight (Mw)[a]
Lipase (from candida rugose)	4.65(-)[b]	58.5 KD
Horseradish peroxidase (HRP)	9.6 (+)[b]	38 KD
Bovine serum albumin (BSA)	5.8 (-)[b]	67 KD
Cytochrome c (Cyt c)	9.1 (+)[b]	13 KD
Glucose oxidase (GOx)	5.0 (-)[b]	65 KD
Myoglobin (MB)	8.7 (+)[b]	17 KD
Trypsin	8.4 (-)[b]	25 KD
Urease	6.0 (-)[b]	90 KD

[a]The parameters were supported by the SIB Bioinformatics Resource Portal, ExPASy (https://www.expasy.org/).

[b]The charge characteristic of the proteins in neutral water solution.

Supplementary Table 2. The protein dosage used and the loading efficiency in the protein@HOF-100

Sample	dosage[a] (mg/mL)	loading[b] (w/w%)
Lipase@HOF-100	0.54	33.7
HRP@HOF-100	0.54	31.8
BSA@HOF-100	0.54	36.6
Cyt c@HOF-100	0.54	35.9
GOx@HOF-100	0.54	36.7
MB@HOF-100	0.54	38.0
Trypsin@HOF-100	0.54	33.3
Urease@HOF-100	0.54	36.1

[a]The dosage (mg/mL) means the protein concentration in the initial assembly system.

[b]The loading (w/w) of Lipase was calculated based on the standard BCA assay and the loading of other proteins were calculated based on the standard Bradford assay.

Supplementary Table 3. Comparison of the protein loading efficiency with other in situ encapsulation strategy using porous organic frameworks

**Biohybrid**	**Proteins**	**Proteins content (w/w, %)**	**Reference**
Proteins@ZIF-8	Cyt c	~8.0	*Nano. Lett*. **2014**, 14, 5761-5765.
Proteins@ZIF-8	GOx, HRP, β-galactosidase, alcohol dehydrogenase	<5	*Nat. Catal*. **2018**,1, 689-695.
Proteins@ZIF-8	HRP, MB	~17.7, ~14.4	*Angew. Chem. Int. Ed*. **2019**, 58, 1463-1467.
Proteins@ZIF-8	HRP, Cyt c, GOx	0.25-4.2	*Angew. Chem. Int. Ed*. **2020**, 59, 13947-13954.
Proteins@ZIF-8	GOx, HRP	8.45, 4.71	*Sci. Adv.* **2020**, *6*, eaax5785.
Proteins@ZIF-8	Cyt c, HRP, CAT, Urate oxidase	0.7-4.2	*Angew. Chem. Int. Ed*. **2020**, 59, 2867-2874.
Proteins@ZIF-90	CAT	6	*J. Am. Chem. Soc*. **2017**,139, 6530-6533.
Proteins@MAF-2[a]	GOx	7.0	*Chem. Eur. J.* **2019** ,25, 5463-5471
Proteins@MAF-7[b]	CAT	3.8, 7.0	*J. Am. Chem. Soc.* **2019**, 141, 2348-2355.
Proteins@UIO-66-NH2[c]	β-glucosidase, invertase, β-galactosidase	∼13.5, ∼14.8, 12.3	*Nat. Commun*. **2019**, 10, 5002.
Enzyme@BioHOF-1	BSA, CAT, AOx	∼6.0	*J. Am. Chem. Soc.* **2019**, 141, 14298−14305
Enzymes@HOF-100	Lipase, HRP, BSA,Cyt c, GOx, MB, trypsin, urease	31.8-38.0	*This work*

[a]MAF-2: Metal azolate frameworks-2, a Cu-MOFs with 3,5 - diethyl - 1,2,4 - triazole as linker

[b]MAF-7: Metal azolate frameworks-7, a Zn-MOFs with 3 - methyl - 1,2,4 - triazole as linker

[c]UIO-66: Zr-MOF with 1,4-benzene-dicarboxylate as linker

Supplementary Table 4. The catalytic kinetic parameters between free Lipase and Lipase@HOF-100

Sample	V_{max} (μM/s)	K_m (μM)
Free Lipase	14.74	485.3
Lipase@HOF-100	12.39	647.8

Supplementary Table 5. Comparison of the analytical performance with other reported biosensors for MUC1 detection

Method	Detection range	Detection limit	Ref.
fluorescence	1 pg/mL - 20 ng/mL	0.23 pg/mL	*Chem. Commun.* 2018, 54, 10195−10198
EC	1 nM -1 μM	0.827 nM	*Anal. Chem.* 2017, 89, 966−973.
DPV	8 pg/ mL-80 ng/mL	2.5 pg/mL	*Biosens. Bioelectron.* 2021, 173, 112785
DPV	1 pg/ mL-50 ng/mL	0.4 pg/mL	*Biosens. Bioelectron.* 2018, 117 474–479
DPV	1 pg/ mL-100 ng/mL	0.18 pg/ mL	This work

Supplementary Table 6. Recovery analysis of MUC1 in Human Serum Samples

Sample number	Detection times	Added MUC1 (pg/mL)	Detected MUC1 (pg/mL)	Recovery (%)	RSD (%)
1	3	1	0.94	94.0	2.53
2	3	10	10.14	101.4	2.50
3	3	100	102.23	102.2	3.43
4	3	1000	1076	107.6	5.81

Supplementary Table 7. The calculated secondary structure of HRP based on CD spectra

HRP in water			HRP in DMF/water[a]		
	Fraction	Ratio		Fraction	Ratio
Helix	0.1	32.8	Helix	0.0	31.1
Beta	0.1	31.6	Beta	0.0	33.7
Turn	0.0	0.0	Turn	0.0	0.0
Random	0.1	35.5	Random	0.0	35.2
Total	0.2	100.0	Total	0.1	100.0
RMS Value	11.224		RMS Value	10.841	

[a]DMF/water solution system contained 300 μL DMF and 9 mL deionized water.

Supplementary Table 8. The calculated secondary structures of GOx and GOx-Pt NCs based on CD spectra

GOx			GOx-Pt NCs		
	Fraction	Ratio		Fraction	Ratio
Helix	0.0	22.2	Helix	0.0	16.6
Beta	0.1	46.0	Beta	0.1	54.4
Turn	0.0	4.9	Turn	0.0	3.9
Random	0.0	26.8	Random	0.0	25.1
Total	0.2	100.0	Total	0.2	100.0
RMS Value	10.956		RMS Value	9.795	

Supplementary Table 9. The calculated secondary structure of Cyt c based on CD spectra

Cyt c in water			Cyt c in DMF/water[a]		
	Fraction	Ratio		Fraction	Ratio
Helix	0.0	33.9	Helix	0.0	34.0
Beta	0.0	10.6	Beta	0.0	7.0
Turn	0.0	24.7	Turn	0.0	26.3
Random	0.0	30.8	Random	0.0	32.8
Total	0.1	100.0	Total	0.1	100.0
RMS Value	12.865		RMS Value	11.722	

[a]DMF/water solution system contained 300 μL DMF and 9 mL deionized water.

Supplementary Table 10. The calculated secondary structure of GOx based on CD spectra

GOx in water			GOx in DMF/water[a]		
	Fraction	Ratio		Fraction	Ratio
Helix	0.0	22.2	Helix	0.0	25.1
Beta	0.1	46.0	Beta	0.0	42.2
Turn	0.0	4.9	Turn	0.0	6.5
Random	0.0	26.8	Random	0.0	26.2
Total	0.2	100.0	Total	0.1	100.0
RMS Value	10.956		RMS Value	8.307	

[a]DMF/water solution system contained 300 μL DMF and 9 mL deionized water.

Supplementary Figures

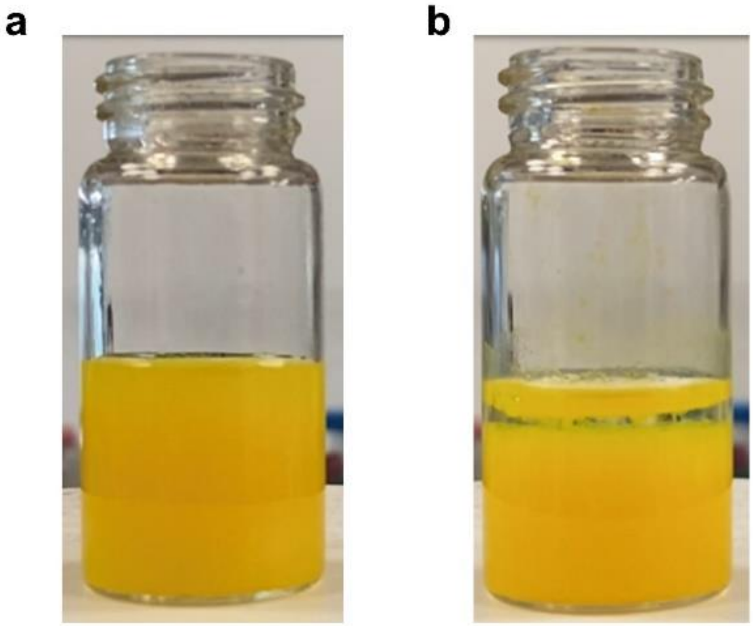

Supplementary Fig. 1. Recording the assembly process by photograph. (a) when HRP was introduced into the H_4TCPy solution; (b) after 5 min reaction.

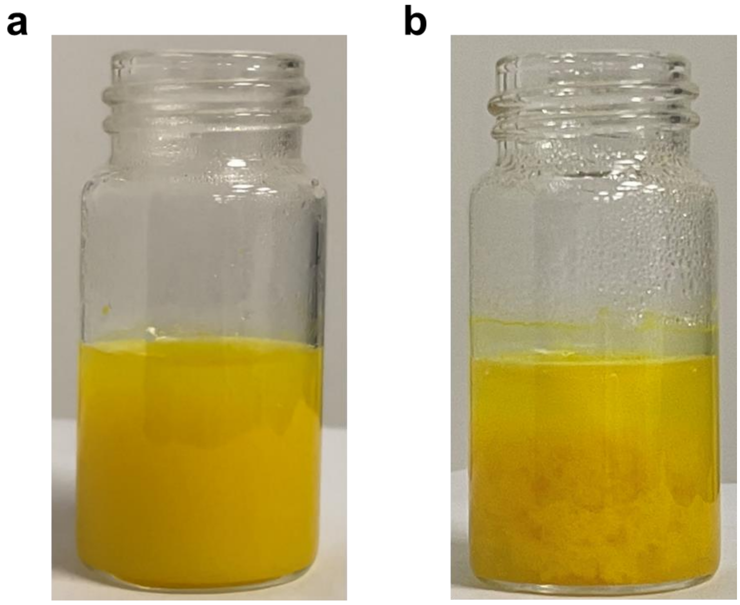

Supplementary Fig. 2. Recording the assembly process by photograph. (a) when H_2O was introduced into the H_4TCPy solution; (b) after 5 min reaction.

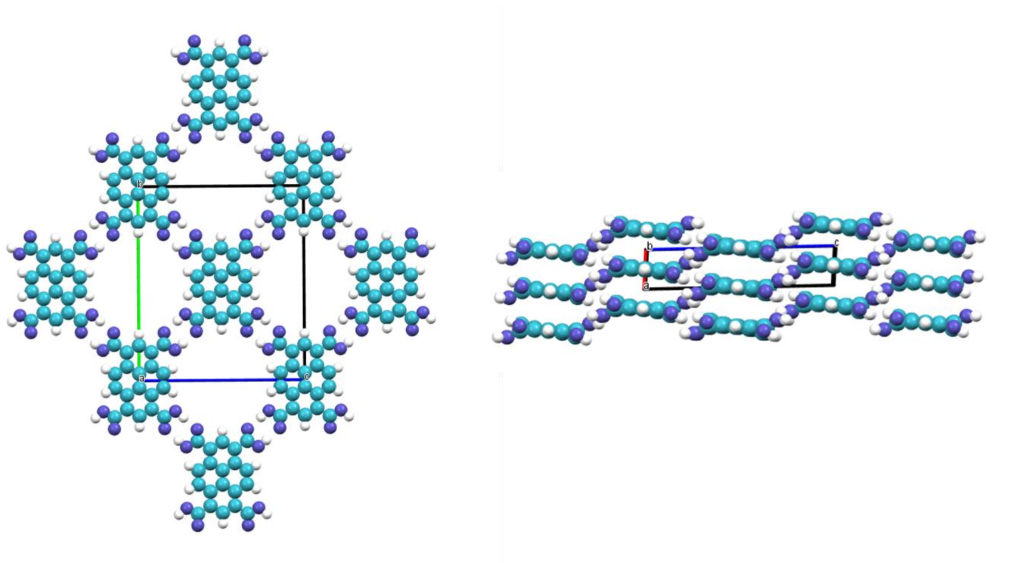

Supplementary Fig. 3. The crystallographic structure of HOF-100. HOF-100 is a recrystallized product of H_4TCPy. The green ball: C atom; the purple ball: O atom; the white ball: H atom.

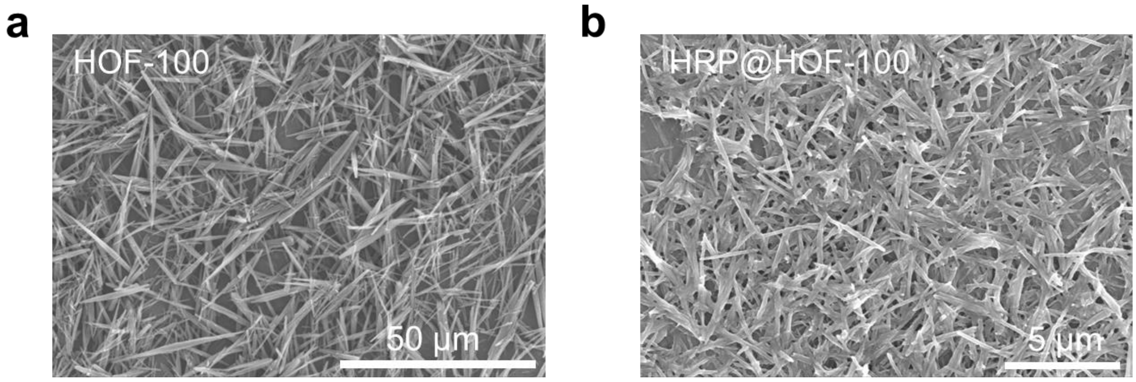

Supplementary Fig. 4. The morphologies of HOF-100 and HRP@HOF-100. SEM images of HOF-100 (a) and HRP@HOF-100 (b). Both of them showed the rod-like nanostructures.

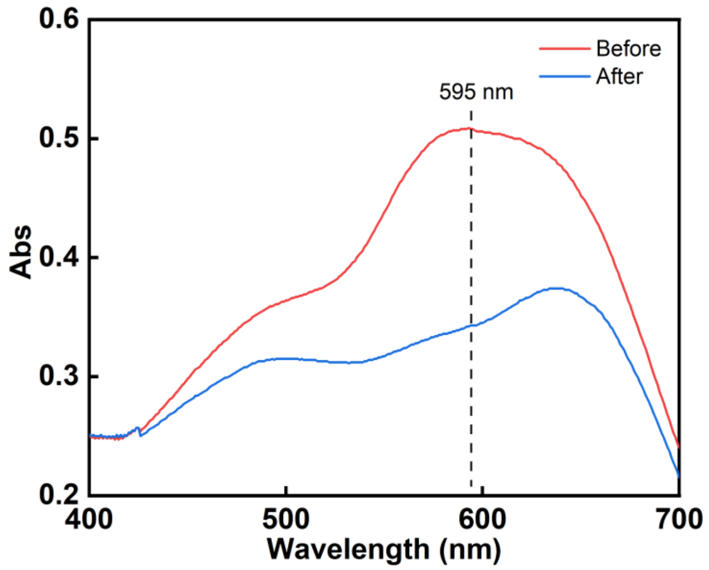

Supplementary Fig. 5. The HRP loading measurement in HRP@HOF-100. The UV-Vis spectra of the standard Bradford protein assay of the collected supernatants before and after encapsulation of HRP by HOF-100. The enzyme concentration in the supernatants was proportion to the UV-Vis absorbance at 595 nm.

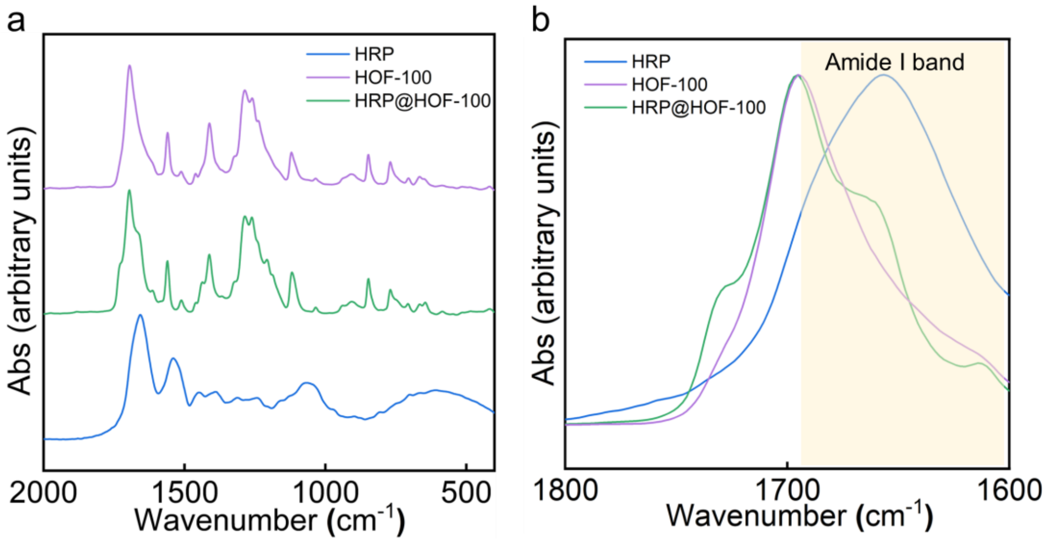

Supplementary Fig. 6. The FT-IR spectra. The full FT-IR spectra (a) and magnifying FT-IR spectra (b) of free HRP, HOF-100 and HRP@HOF-100. The occurrence of spectral band (amide I, 1700−1610 cm^{-1}) in the synthesized HRP@HOF-100 was designated as the emblematic peptide skeleton of the enzyme, attesting the successful encapsulation of enzymes.

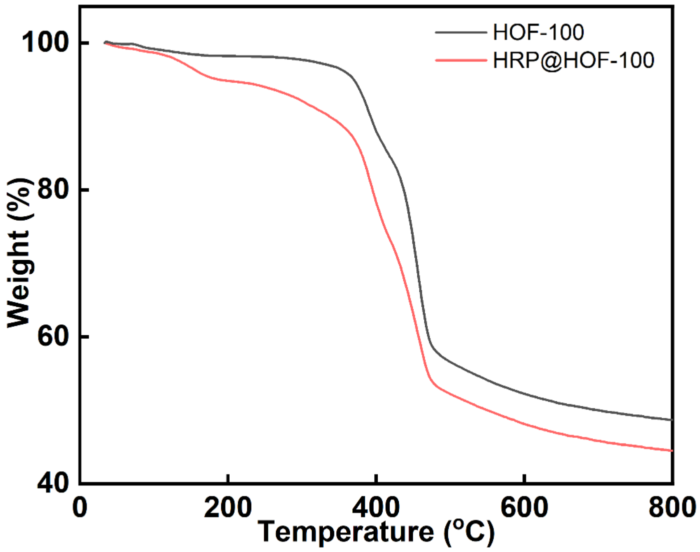

Supplementary Fig. 7. The TGA analysis. TGA analysis of HRP@HOF-100 and pure HOF-100. The weight loss at ca. 200-350 °C was caused by the pyrolysis of HRP, evidencing the encapsulation of HRP.

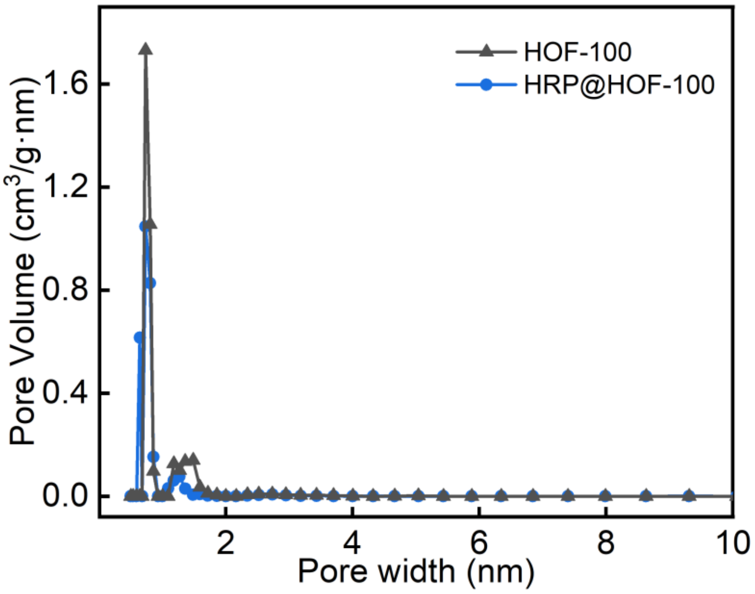

Supplementary Fig. 8. The pore-size distributions of HOF-100 and HRP@HOF-100. The pore-size distributions of HOF-100 and HRP@HOF-100, both of them showed the microporous structure. Compared to HOF-100, HRP@HOF-100 showed a deceased pore volume. This was caused by the spatial occupation by the enzymes, which further demonstrated that the enzymes were indeed encapsulated into, rather than surface-adsorbed onto the HOF-100.

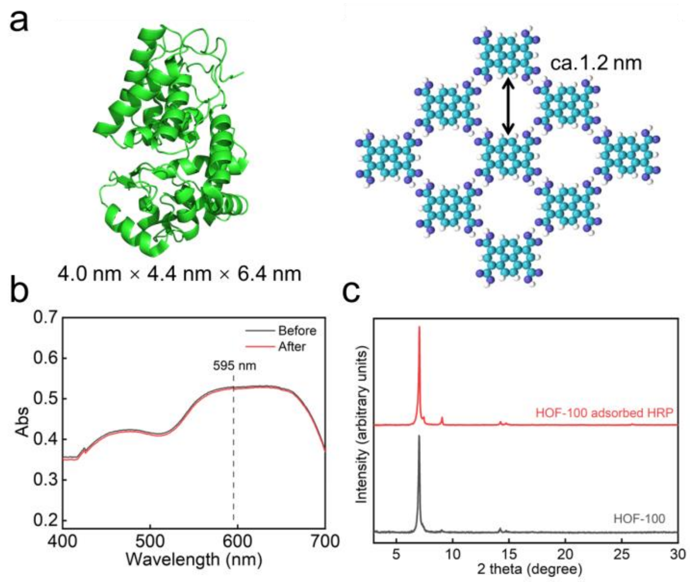

Supplementary Fig. 9. The surface-adsorption experiment. (a) The molecular dimension of HRP and the crystallographic pore of HOF-100. The colors used in molecular tectons of H_4TCPy are: green for C atom; violet for O atom; white for H. (b) The UV-Vis spectra of the standard Bradford protein assay of the supernatants before and after adsorption experiment. The enzyme concentration was proportion to the UV-Vis absorbance at 595 nm. (c) PXRD patterns of HOF-100 before and after HRP adsorption.

Note: Theoretically, the bulky HRP (ca. 4.0 nm × 4.4 nm × 6.4 nm, PDB: 1hch) could not pass through the relatively narrow micropore of HOF-100 (Supplementary Fig. 9a). It indicated that the HRP was encapsulated into the framework in situ. To confirm this, 5 mg HRP was dispersed in 9 mL of as-synthesized HOF-100 solution (1 mg/mL). After stirring for 5 min and standing for 15 min, the HRP-adsorbed HOF-100 was collected by centrifugation. The surface-adsorbed enzymes by HOF-100 were evaluated based on the concentration change of the enzymes in the supernatants before and after adsorption using standard Bradford assay. The UV-Vis spectra of the collected supernatants showed that almost no enzyme was adsorbed by HOF-100 (Supplementary Fig. 9b). In addition, the HOF-100 retained intact crystallinity after the adsorption experiment (Supplementary Fig. 9c), suggesting that the HRP adsorption experiment could not affect the crystallographic structure of HOF.

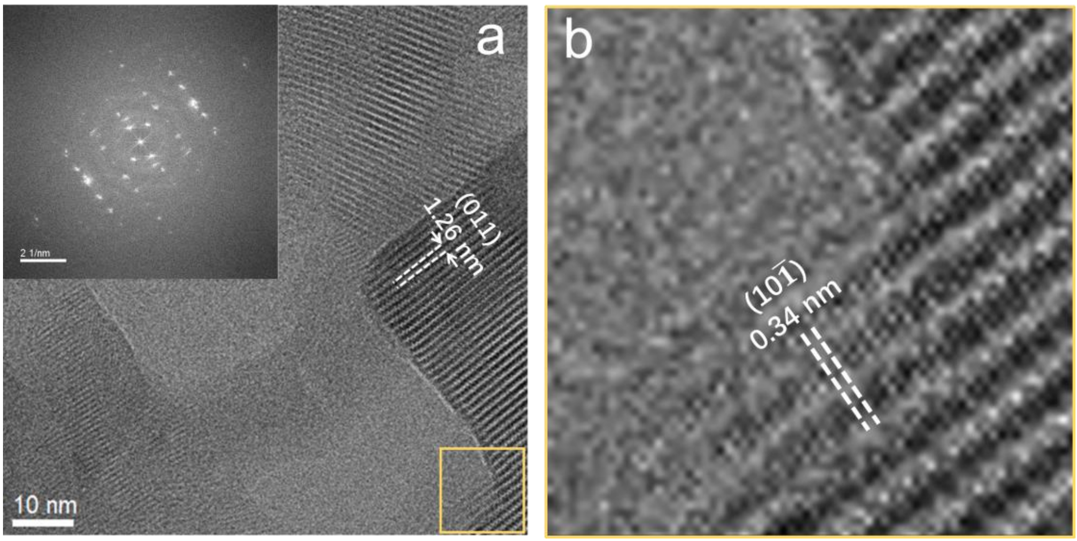

Supplementary Fig. 10. Cryo-EM images of HRP@HOF-100. (a) The cryo-EM image showing the long-range ordered channels within HRP@HOF-100 biocatalyst. The inset was the fast Fourier transform image. (b) The enlarged domain highlighted in yellow in (a).

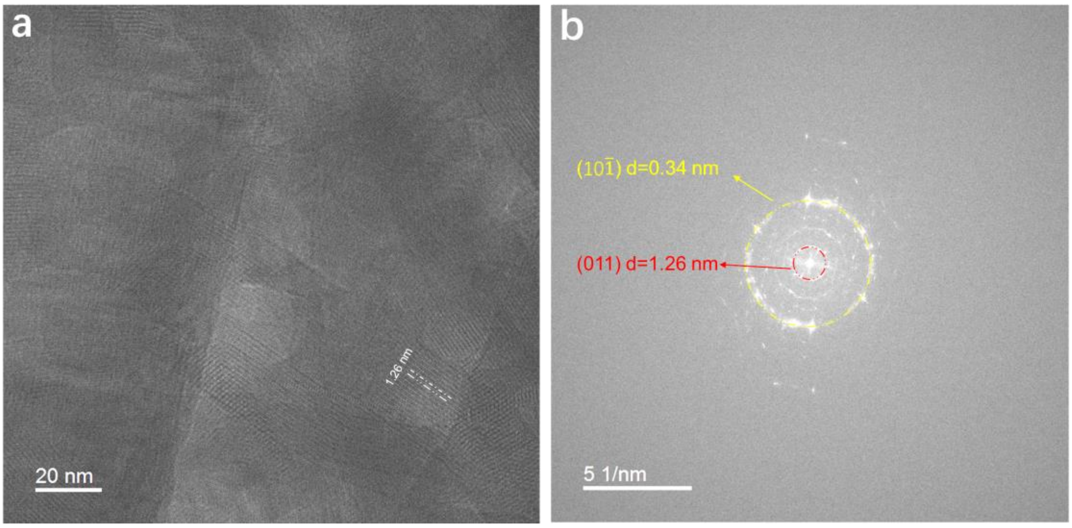

Supplementary Fig. 11. Large-area cryo-EM image of HRP@HOF-100. (a) The cryo-EM image showing the large area of HRP@HOF-100 biocatalyst. (b) The corresponding FFT image of (a), in which the (011) and (10$\bar{1}$) lattice planes were highlighted.

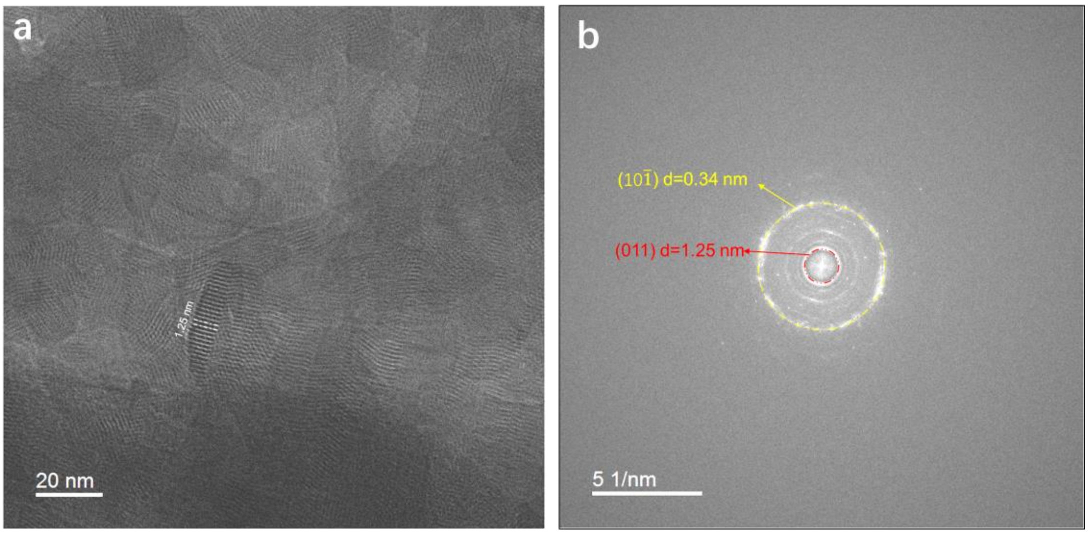

Supplementary Fig. 12. Large-area cryo-EM image of HRP@HOF-100. (a) The cryo-EM image showing the large area of HRP@HOF-100 biocatalyst. (b) The corresponding FFT image of (a), in which the (011) and $(10\bar{1})$ lattice planes were highlighted.

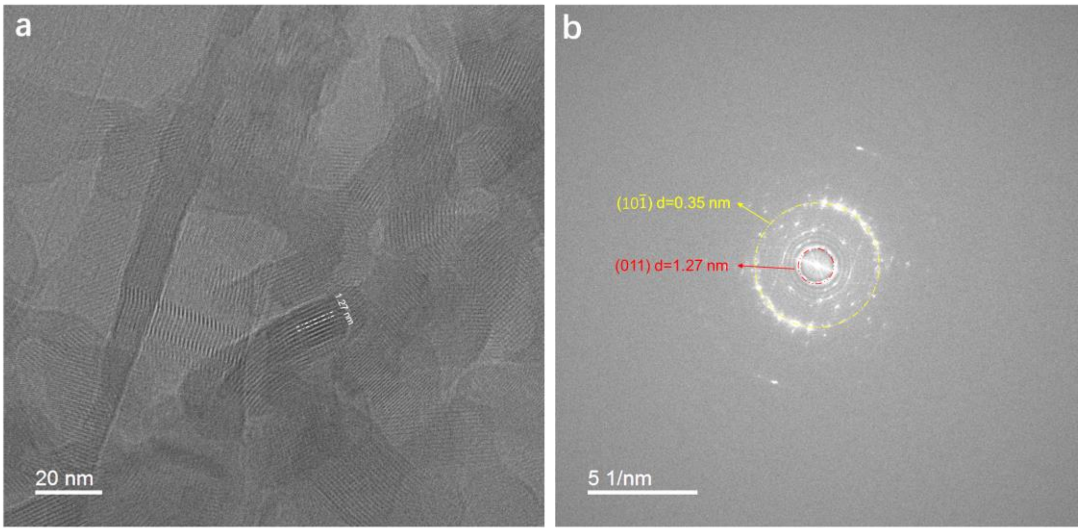

Supplementary Fig. 13. Large-area cryo-EM image of HRP@HOF-100. (a) The cryo-EM image showing the large area of HRP@HOF-100 biocatalyst. (b) The corresponding FFT image of (a), in which the (011) and $(10\bar{1})$ lattice planes were highlighted.

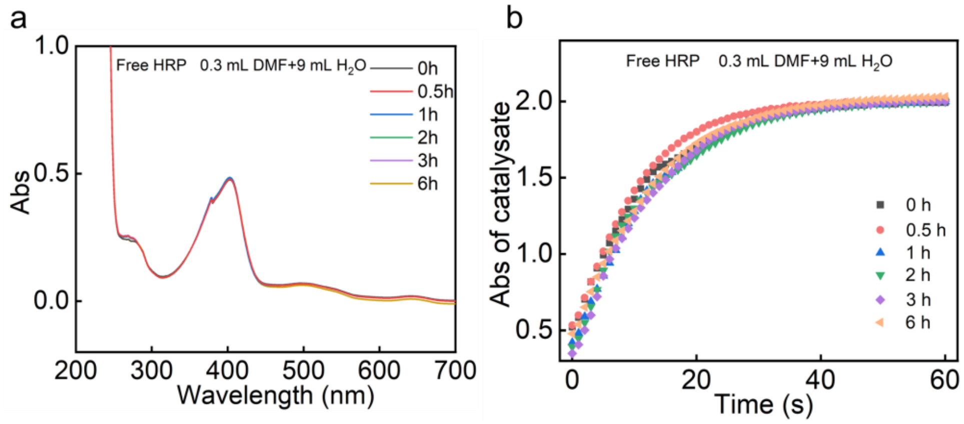

Supplementary Fig. 14. The conformation and catalytic activity of HRP after incubating in aqueous solution involving minute amount of DMF. The UV-Vis spectra (a) and catalytic kinetics curves (b) of free HRP after incubating in DMF aqueous solution for different times. The volumes of DMF and deionized water were 0.3 mL and 9.0 mL, respectively, which were in agreement with the assembling condition of HRP@HOF-100 biocatalyst.

Note: The intact UV-Vis spectra confirmed that the DMF aqueous solution adopted in our encapsulation procedure could not affect the conformation of HRP. The unchanged catalytic kinetics curves indicated the HRP could well reserve its bioactivity after exposing to the DMF aqueous solution.

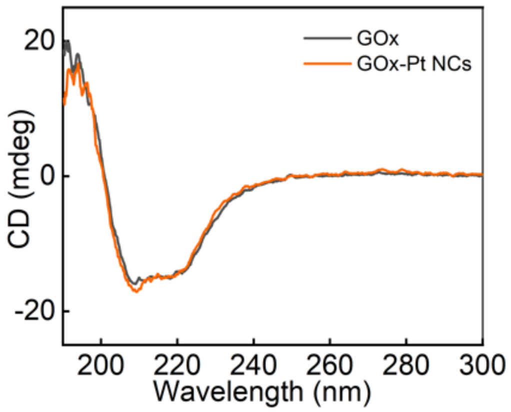

Supplementary Fig. 15. The CD spectra. The CD spectra of free GOx and GOx-Pt NCs. The calculated secondary structure contents based on Yang's reference are displayed in Supplementary Table 8.

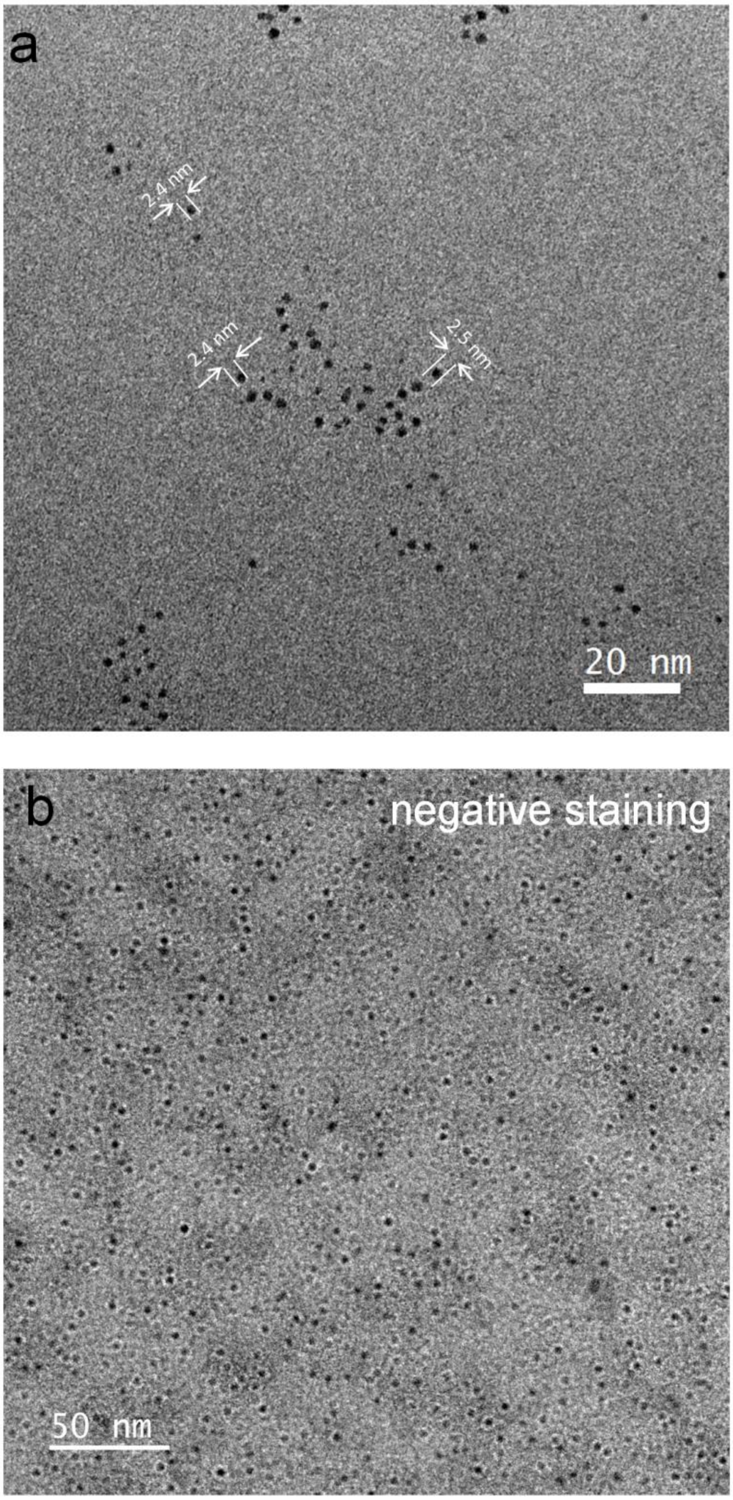

Supplementary Fig. 16. The TEM images of GOx-Pt NCs before and after negative staining. (a) The TEM image of GOx-Pt NCs. (b) The TEM image of GOx-Pt NCs after the protein negative staining treatment. Under the protein negative staining experiment, we clearly observed the enzyme outline (in white) around individual Pt NCs, suggesting each enzyme was successfully labelled by a Pt NCs.

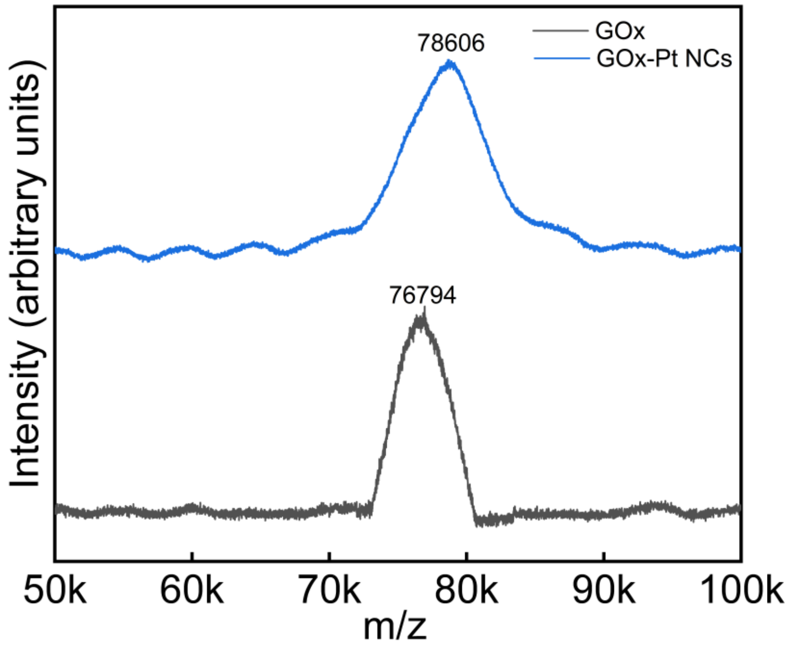

Supplementary Fig. 17. The MALDI-TOF analysis. MALDI-TOF analysis of the molecular weight of GOx and GOx-Pt NCs. The insight into the molecular weight MALDI-TOF MS showed that the enzyme weight increased from 76794 to 78606 after labeling, and it gave a labelling efficiency of ca. nine Pt per one enzyme.

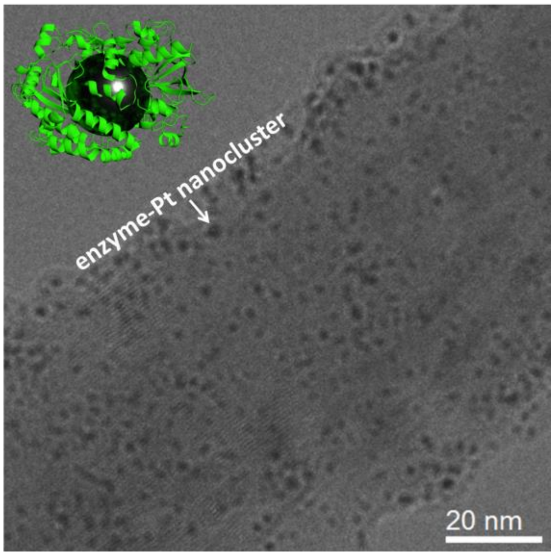

Supplementary Fig. 18. The cryo-EM image of GOx-Pt@HOF-100. Low-magnification cryo-EM image of GOx-Pt NCs-encapsulated HOF-100.

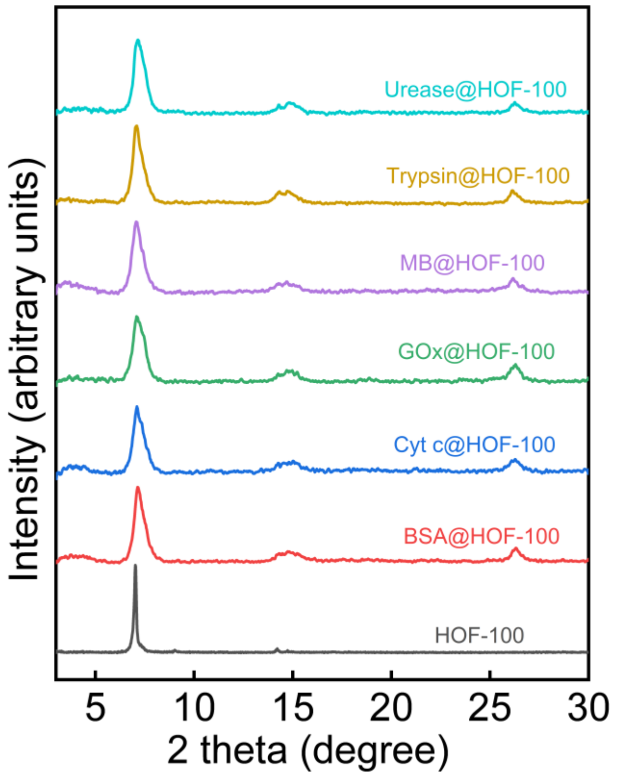

Supplementary Fig. 19. The crystallinities of proteins@HOF-100. The PXRD patterns of the synthesized proteins@HOF-100 using different proteins including BSA, Cyt c, GOx, MB, trypsin and urease.

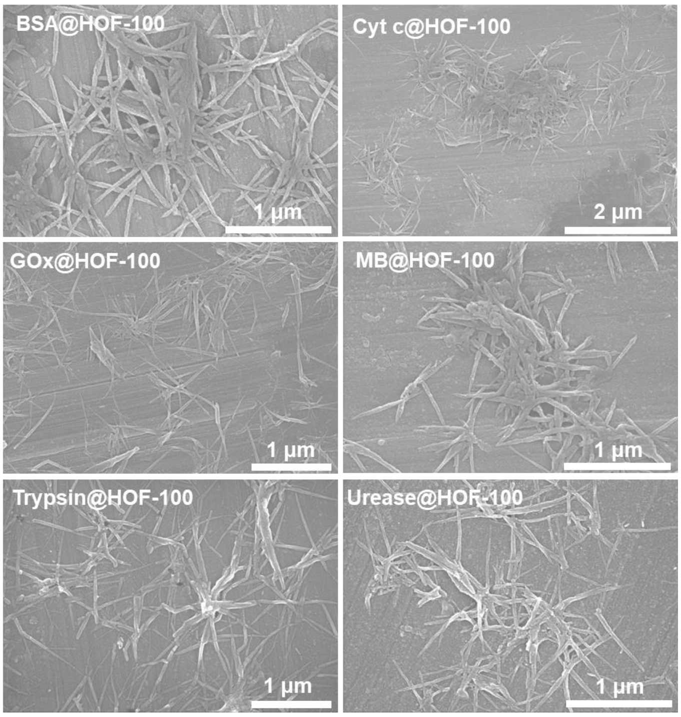

Supplementary Fig. 20 The morphologies of proteins@HOF-100. SEM images of the synthesized proteins@HOF-100 using different proteins including BSA, Cyt c, GOx, MB, trypsin and urease. The SEM images presented the rod-like nanostructures of the as-synthesized proteins@HOF-100, which were in great agreement with that of the parent HOF-100.

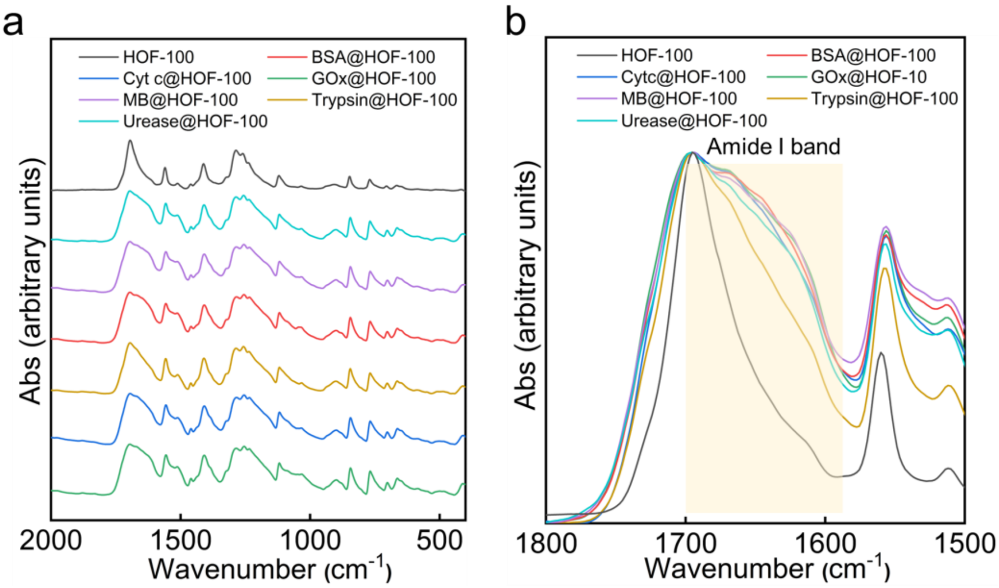

Supplementary Fig. 21. The FT-IR spectra. The full FT-IR spectra (a) and magnifying FT-IR spectra (b) of the synthesized proteins@HOF-100 using different proteins including BSA, Cyt c, GOx, MB, trypsin and urease. The occurrences of spectral band (amide I, 1700−1610 cm^{-1}) in the synthesized proteins@HOF-100 were designated as the emblematic peptide skeleton of the proteins, attesting the successful encapsulation of proteins.

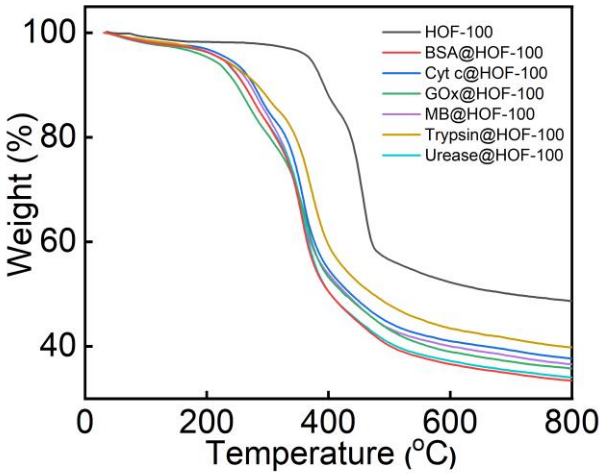

Supplementary Fig. 22. The TGA analysis. TGA analysis of the as-synthesized proteins@HOF-100 using different proteins including BSA, Cyt c, GOx, MB, trypsin and urease. The weight loss at ca. 200-350 °C was caused by the pyrolysis of proteins, evidencing the encapsulation of proteins.

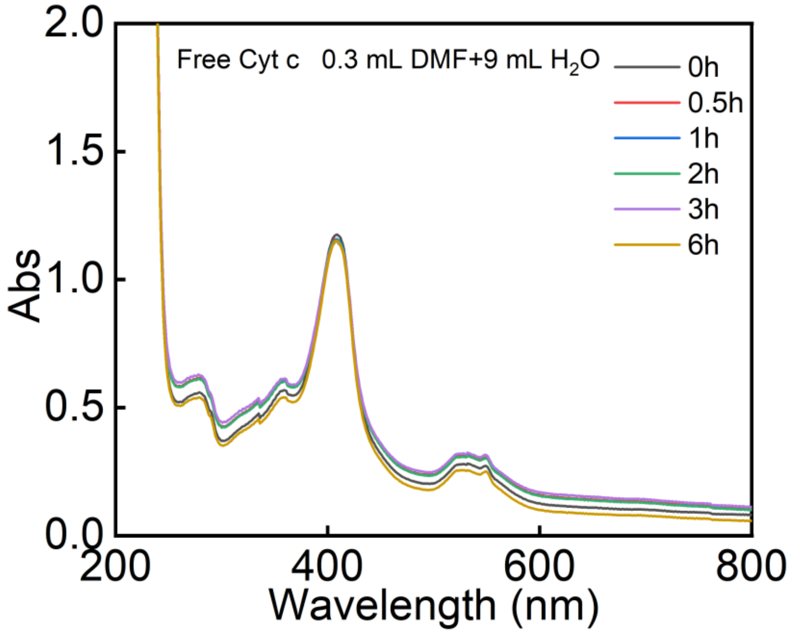

Supplementary Fig. 23. The UV-Vis spectra of Cyt c after incubating in aqueous solution involving minute amount of DMF. The UV-Vis spectra of free Cyt c after incubating in DMF aqueous solution for different times. The volumes of DMF and deionized water were 0.3 mL and 9.0 mL, respectively, which were in agreement with the ones used in Cyt c@HOF-100 synthesis.

Note: Cyt c has a ferriporphyrin active center. The microenvironments of the active center of Cyt c could be profiled by UV-Vis spectroscopy, a sensitive means for heme coordination study.[8] As shown in Supplementary Fig. 23, when Cyt c was exposed in the DMF aqueous solution (containing 9 mL of water and 0.3 mL of DMF), the UV-Vis adsorption profile of Cyt c was well retained even after 6 h. This experiment suggested that the DMF aqueous solution adopted in our encapsulation procedure is biocompatible to Cyt c.

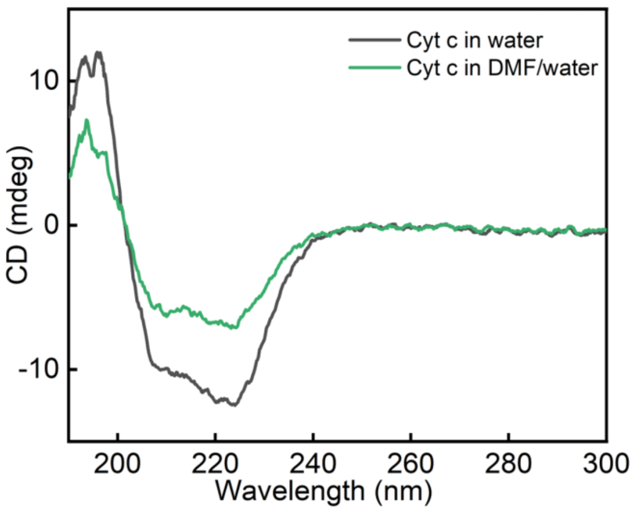

Supplementary Fig. 24. The CD spectra. The CD spectra of free Cyt c in water and Cyt c after incubating in DMF (300 μL) aqueous solution for 6 h. The calculated secondary structure contents of Cyt c based on Yang's reference are displayed in Supplementary Table 9. The volumes of DMF and deionized water were 0.3 mL and 9.0 mL, respectively, which were in agreement with the ones used in Cytc@HOF-100 synthesis. This result indicated that the conformation of enzyme could be well preserved even after 6 h.

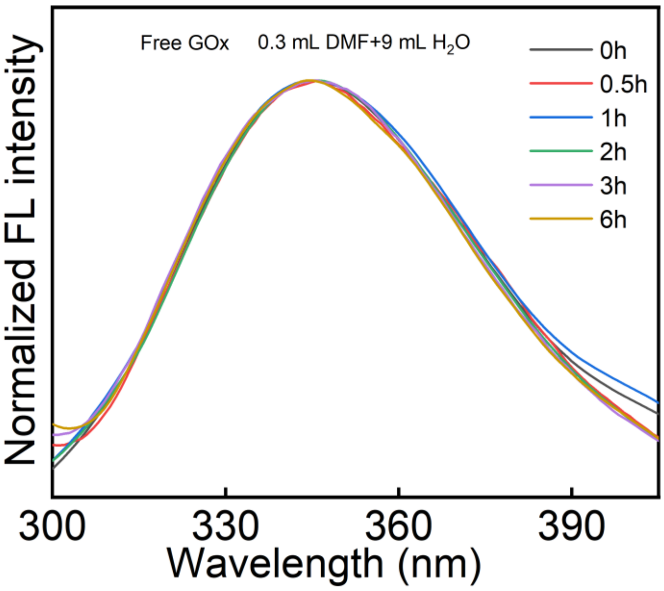

Supplementary Fig. 25. The fluorescence spectroscopy of GOx after incubating in aqueous solution involving minute amount of DMF. The fluorescence spectroscopy of free GOx after incubating in DMF aqueous solution for different times. The volumes of DMF and deionized water were 0.3 mL and 9.0 mL, respectively, which were in agreement with the ones used in GOx@HOF-100 synthesis.

Note: GOx has limited UV-Vis adsorption bands, and thus UV-Vis spectroscopy is unable to investigate the conformation of GOx. The fluorescence emission profile of enzyme depends on the structural conformation of buried amino acids, and thus fluorescence spectroscopy could be used to monitor the structural changes of the enzyme.[9] As shown in Supplementary Fig. 25, when GOx was exposed in the DMF aqueous solution (containing 9 mL of water and 0.3 mL of DMF), the fluorescence emission profile of GOx was well retained even after 6 h. This experiment suggested that the DMF aqueous solution adopted in our encapsulation procedure was biocompatible to GOx.

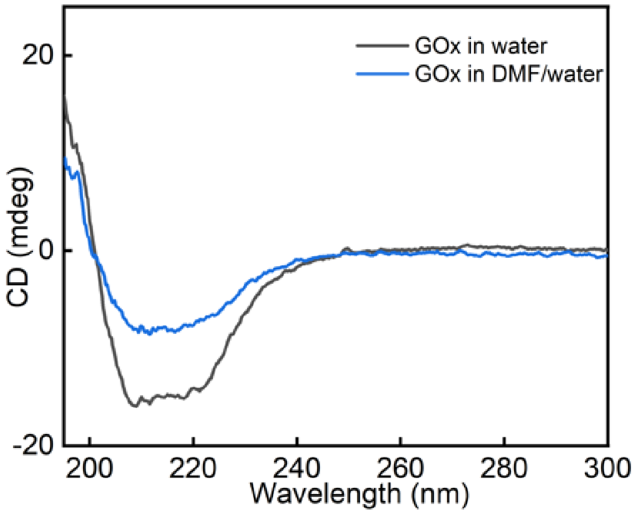

Supplementary Fig. 26. The CD spectra. The CD spectra of free GOx in water and the GOx after incubating in DMF (300 μL) aqueous solution for 6 h. The calculated secondary structure contents of GOx based on Yang's reference were displayed in Supplementary Table 10. The volumes of DMF and deionized water were 0.3 mL and 9.0 mL, respectively, which were in agreement with the ones used in GOx@HOF-100 synthesis. This result indicated that the conformation of enzyme could be well preserved even after 6 h.

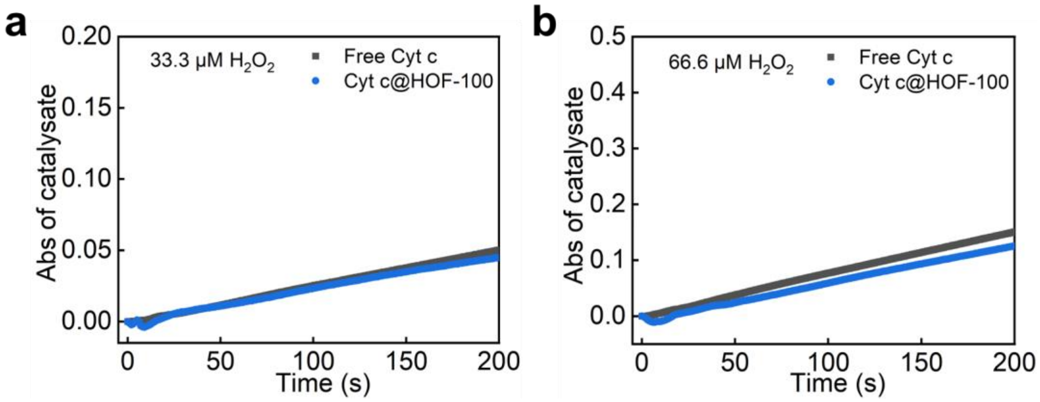

Supplementary Fig. 27. The catalytic kinetics curves. The catalytic kinetics curves of free Cyt c and Cyt c@HOF-100 under 33.3 μM (a) and 66.6 μM (b) H_2O_2 concentrations. The dosages of Cyt c (33.3 μg/mL) used were kept the same in each trial.

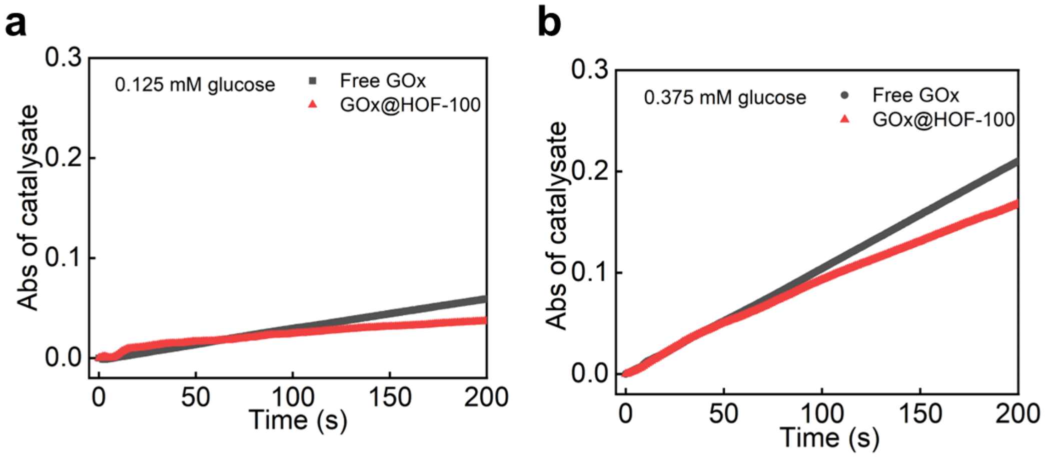

Supplementary Fig. 28. The catalytic kinetics curves. The catalytic kinetics curves of free GOx and GOx@HOF-100 under 0.125 mM (a) and 0.375 mM (b) glucose concentrations. The dosages of GOx (15 μg/mL) used were kept the same in each trial.

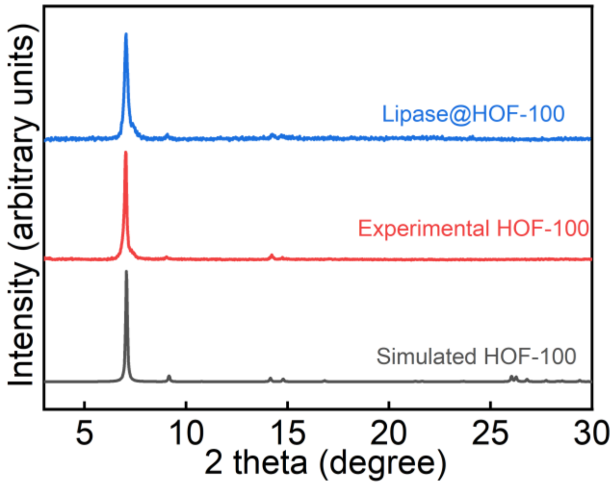

Supplementary Fig. 29. The crystallinity of Lipase@HOF-100. The PXRD patterns of Lipase@HOF-100, experimental HOF-100 and simulated HOF-100.

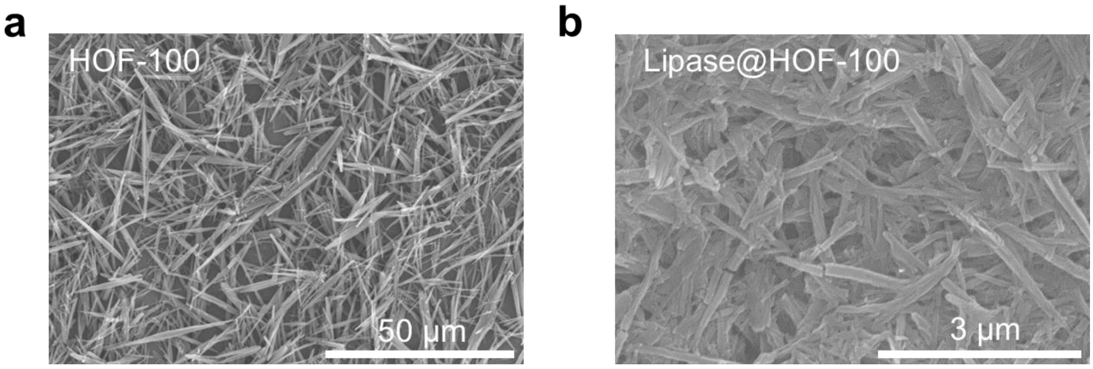

Supplementary Fig. 30. The morphologies of HOF-100 and Lipase@HOF-100. SEM images of HOF-100 (a) and Lipase@HOF-100 biocatalyst (b). Both of them showed rod-like nanostructures.

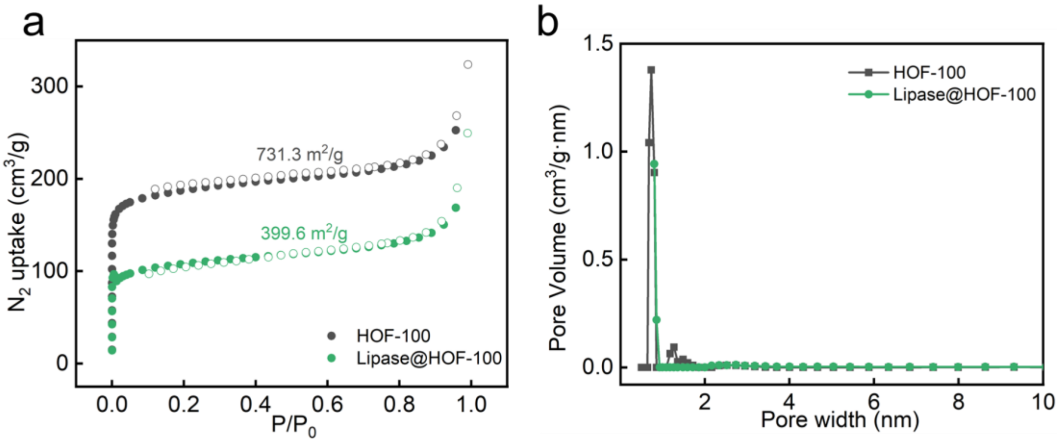

Supplementary Fig. 31. The nitrogen adsorption/desorption isotherms and pore-size distributions of HOF-100 and Lipase@HOF-100. (a) Nitrogen adsorption/desorption isotherms of Lipase@HOF-100 and pure HOF-100. (b) The pore-size distributions of HOF-100 and Lipase@HOF-100 based on NLDFT method. Lipase@HOF-100 featured a typical I-type curves with a calculated BET surface area of 399.6 m^2/g. Nevertheless, this BET value was reduced compared to the pure HOF-100 (731.3 m^2/g), manifesting that the interior pore of HOF-100 was partially occupied by Lipase. (b) The pore-size distributions of HOF-100 and Lipase@HOF-100 based on NLDFT method. Both them showed the microporous structure. Compared to HOF-100, Lipase@HOF-100 showed a deceased pore volume. This was caused by the spatial occupation by the enzymes, which further demonstrated that the enzymes were indeed encapsulated into, rather than surface-adsorbed onto the HOF-100.

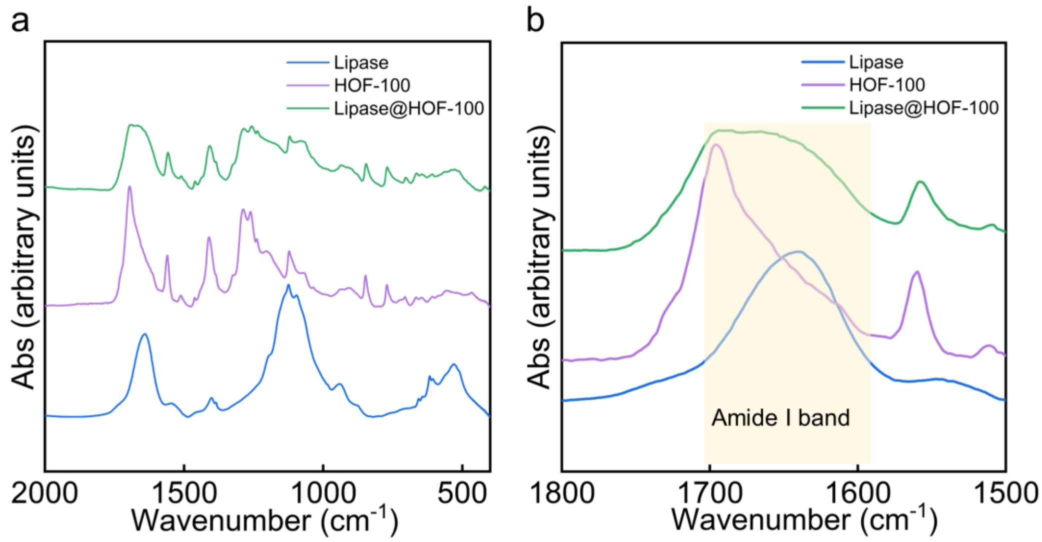

Supplementary Fig. 32. The FT-IR spectra. The full FT-IR spectra (a) and magnifying FT-IR spectra (b) of the synthesized Lipase@HOF-100. The occurrence of spectral band (amide I, 1700−1610 cm^{-1}) in the synthesized Lipase@HOF-100 was designated as the emblematic peptide skeleton of the enzyme, attesting the successful encapsulation of enzymes.

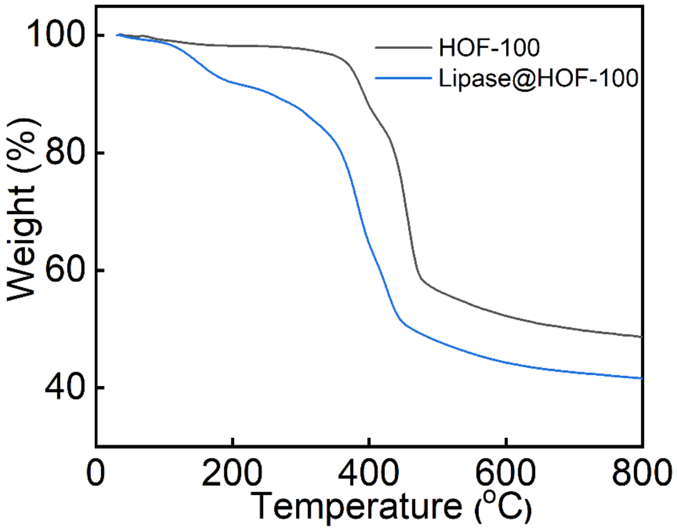

Supplementary Fig. 33. The TGA analysis. TGA analysis of Lipase@HOF-100 and pure HOF-100. The weight loss at ca. 200-350 °C was caused by the pyrolysis of Lipase, evidencing the encapsulation of Lipase.

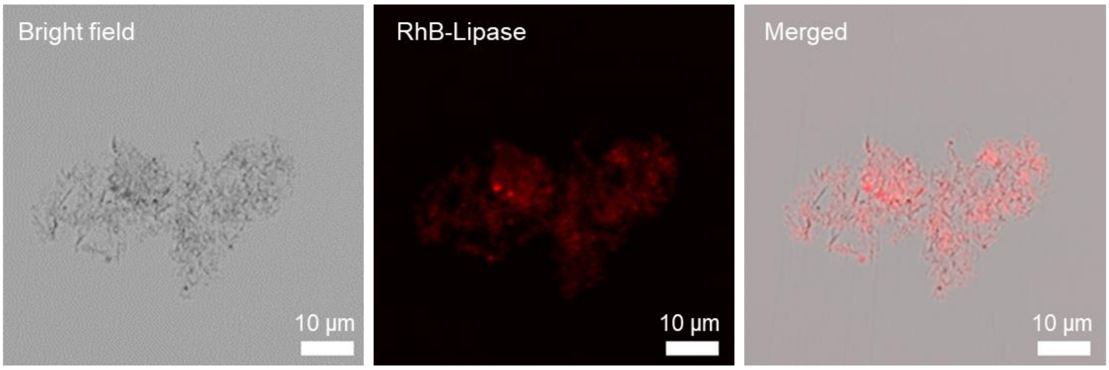

Supplementary Fig. 34. The spatial distribution of Lipase within HOF-100. The CLSM images showing the spatial distribution of Lipase (Lipase was labelled by red dye) within HOF-100 scaffold.

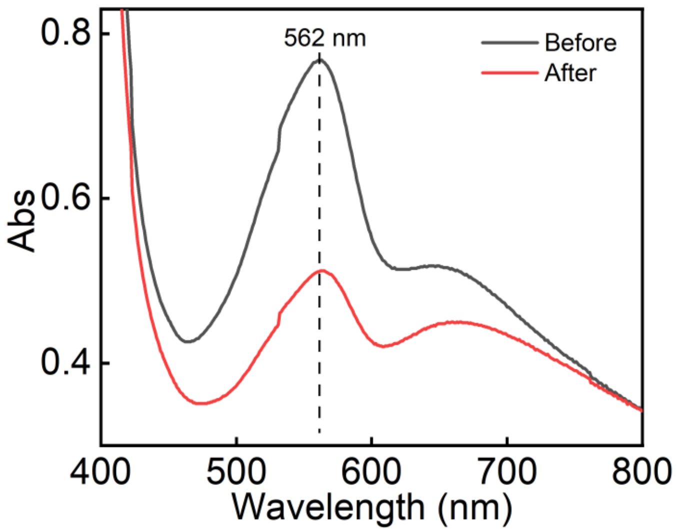

Supplementary Fig. 35. The Lipase loading measurement of Lipase@HOF-100. The UV-Vis spectra of the standard BCA protein assay of the collected supernatants before and after encapsulation of Lipase by HOF-100. The enzyme concentration in the supernatants was proportion to the UV-Vis absorbance at 562 nm.

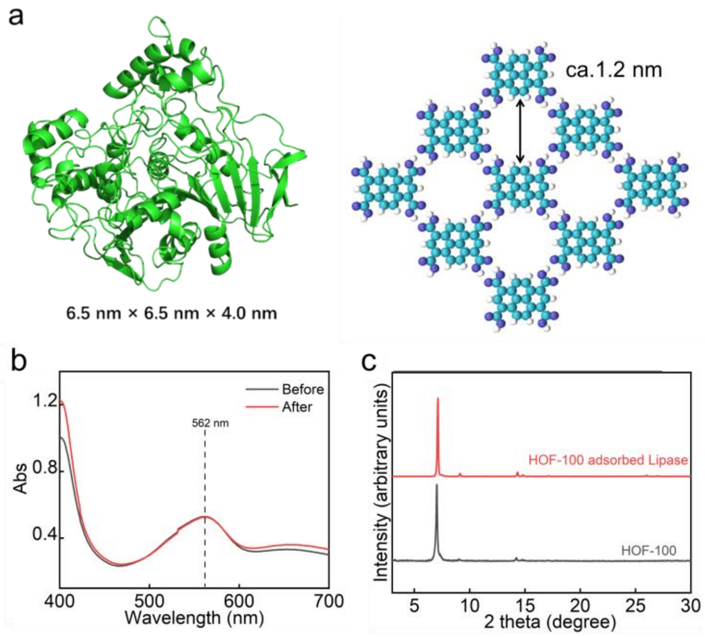

Supplementary Fig. 36. The surface-adsorption experiment of Lipase. (a) The molecular dimension of Lipase and the crystallographic pore of HOF-100. The colors used in molecular tectons of H_4TCPy are: green for C atom; violet for O atom; white for H. (b) The UV-Vis spectra of the standard BCA protein assay of the supernatants before and after adsorption experiment. The Lipase concentration was proportion to the UV-Vis absorbance at 562 nm. (c) PXRD patterns of HOF-100 before and after Lipase adsorption.

Note: Theoretically, the bulky Lipase cannot pass through the relatively narrow micropore of HOF-100 (Supplementary Fig. 36a, the molecular dimension of Lipase (PDB: 1trh) is ca. 6.5 nm × 6.5 nm × 4.0 nm, which was measured by PyMOL). To confirm this, 5 mg Lipase was dispersed in 9 mL of as-synthesized HOF-100 solution (1 mg/mL). After stirring for 5 min and standing for 15 min, the Lipase-adsorbed HOF-100 was collected by centrifugation. The surface-adsorbed enzymes by HOF-100 were evaluated based on the concentration change of the enzymes in the supernatants before and after adsorption using standard BCA assay. The UV-Vis spectra of the collected supernatants showed that almost no enzyme was adsorbed by HOF-100 (Supplementary Fig. 36b). In addition, the HOF-100 retained intact crystallinity after the adsorption experiment (Supplementary Fig. 36c), suggesting that the Lipase adsorption experiment could not affect the crystallographic structure of HOF-100.

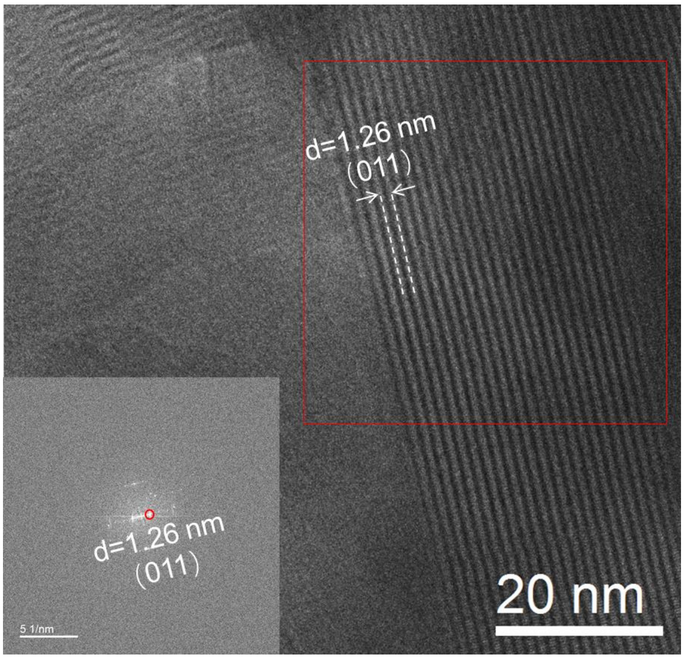

Supplementary Fig. 37. cryo-EM image of Lipase@HOF-100. The cryo-EM image showing the long-range ordered channels within Lipase@HOF-100.

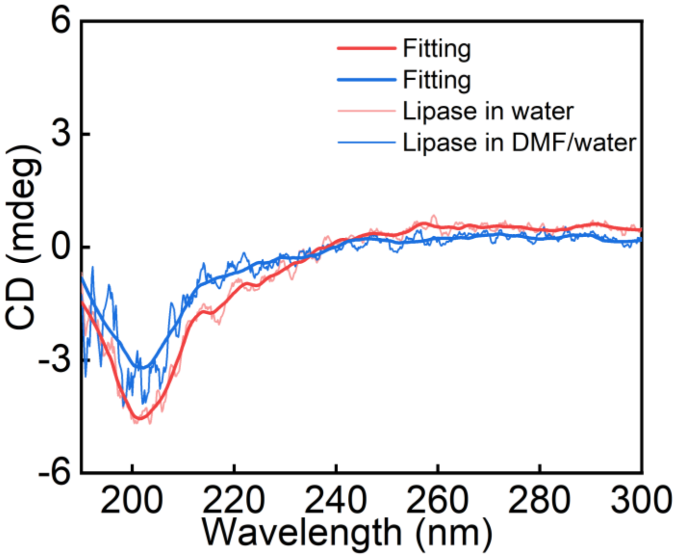

Supplementary Fig. 38. The CD spectra. The CD spectra of free Lipase in water and the Lipase after incubating in DMF aqueous solution for 6 h. The volumes of DMF and deionized water were 0.3 mL and 9.0 mL, respectively, which were in agreement with the ones used in the Lipase@HOF-100 synthesis. This result indicated that the conformation of Lipase could be well preserved even after 6 h.

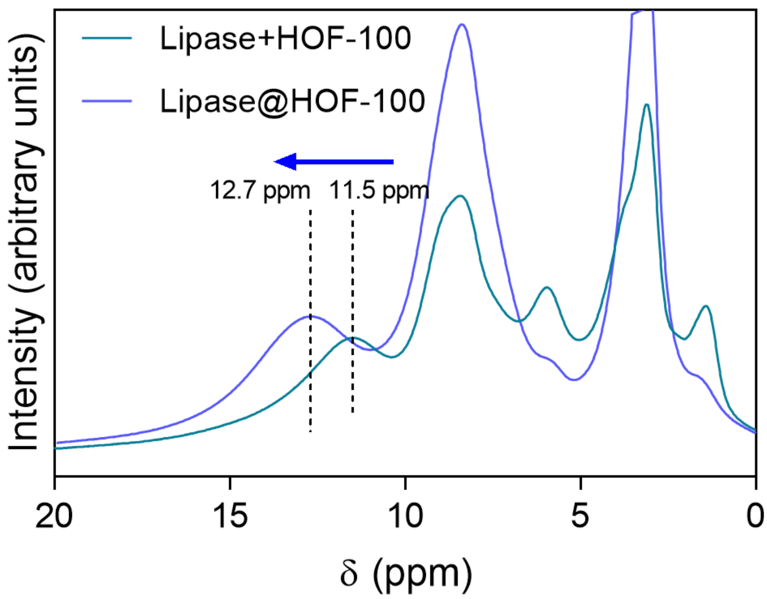

Supplementary Fig. 39. The H^1 ssNMR spectra of HOF-100 and Lipase@HOF-100. The ssNMR spectra of Lipase@HOF-100 biocomposite and the physically mixed sample of Lipase and HOF-100.

Note: In the as-synthesized Lipase@HOF-100, the biointerface between Lipase and HOF-100 was firstly examined by solid-state nuclear magnetic resonance (ssNMR, Supplementary Fig. 39). The chemical shift at 12.7 ppm in H^1 ssNMR of Lipase@HOF-100 biocatalyst is assigned to the proton of the carboxyl groups of H_4TCPy molecular tectons. This peak is observed to be shifted into a low magnetic field compared with the physical mixed sample of Lipase and HOF-100 (δ=11.5 ppm), suggesting the biointerface interaction between Lipase and the carboxyl groups of HOF-100 scaffold.

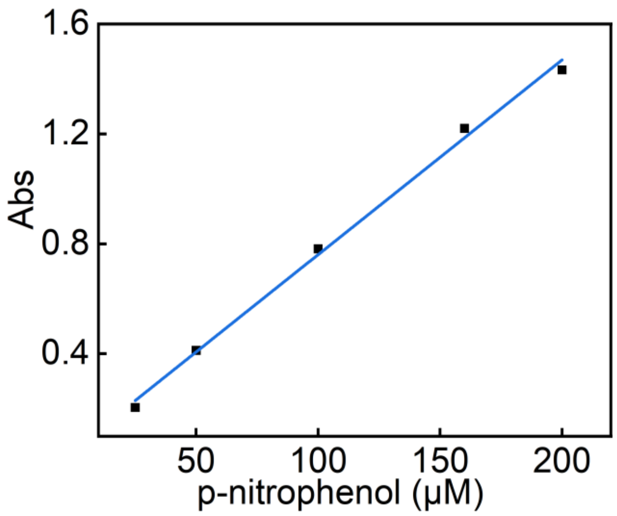

Supplementary Fig. 40. The calibration curve of p-nitrophenol. The p-nitrophenol calibration curve for the bioactivity measurement of Lipase and Lipase@HOF-100.

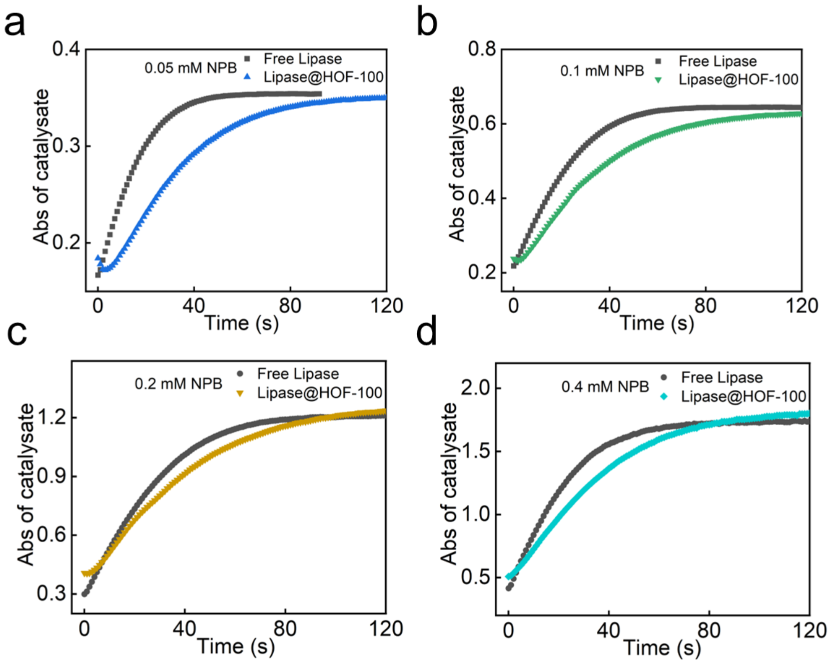

Supplementary Fig. 41. The catalytic kinetics curves. The catalytic kinetics curves of free Lipase and Lipase@HOF-100 under 0.05 mM (a), 0.1 mM (b), 0.2 mM (c) and 0.4 mM (d) NPB concentrations. The dosages of Lipase (80 μg/mL) used were kept the same in each trial. The decreases in the initial few seconds of Lipase@HOF-100 was caused by the transitory change in transmittance after adding the substrate solution into HOF particles.

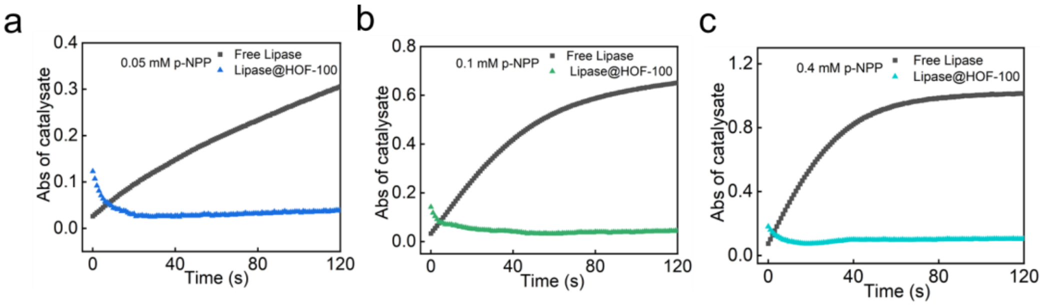

Supplementary Fig. 42. The catalytic kinetics curves. The catalytic kinetics curves of free Lipase and Lipase@HOF-100 under 0.05 mM (a), 0.1 mM (b) and 0.4 mM (c) p-NPP concentrations. The dosages of Lipase (80 µg/mL) used were kept the same in each trial. The decreases in the initial few seconds of Lipase@HOF-100 was caused by the transitory change in transmittance after adding the substrate solution into HOF particles.

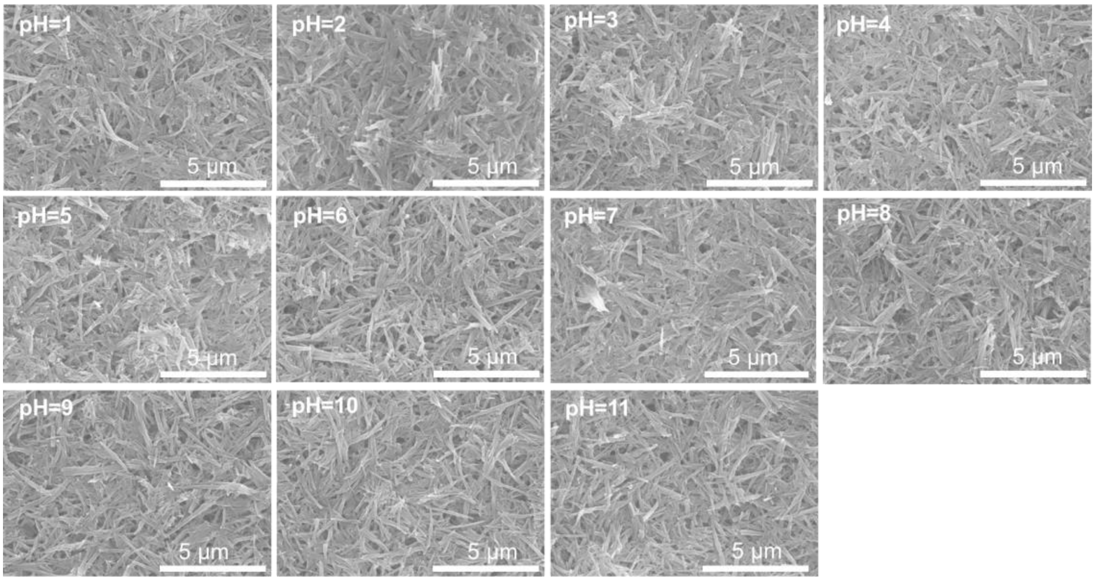

Supplementary Fig. 43. The morphologies of Lipase@HOF-100 after different pH treatments. SEM images of Lipase@HOF-100 after different pH treatments for 30 min. **Note:** After exposing the as-prepared Lipase@HOF-100 to aqueous solutions with different pH for 30 min, all of the treated Lipase@HOF-100 maintained rod-like nanostructures. In these exposure experiment, 100 µL of Lipase@HOF-100 dispersive solution (4 mg/mL) was mixed with 900 µL of aqueous solution of different pH, following by standing for 30 min. Both the intact PXRD patterns (Fig. 5b in main text) and SEM images (Supplementary Fig. 43) demonstrated the highly structural stability of Lipase@HOF-100.

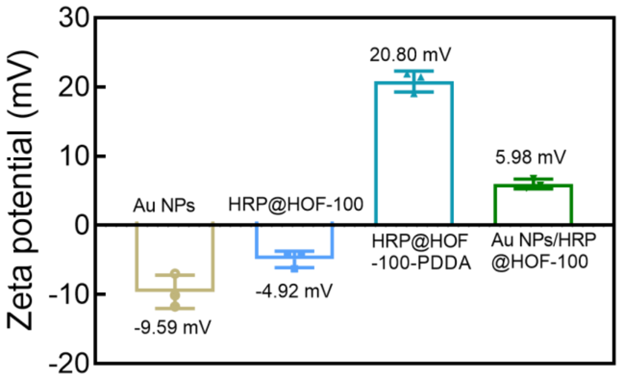

Supplementary Fig. 44. The Zeta potentials. The Zeta potentials of Au NPs, HRP@HOF-100, HRP@HOF-100-PDDA and Au NPs/HRP@HOF-100. The data of 3 independent experiments and calculated error bars (SD) are presented, SD = Standard Deviation (n = 3). Data are presented as mean values +/- SD.

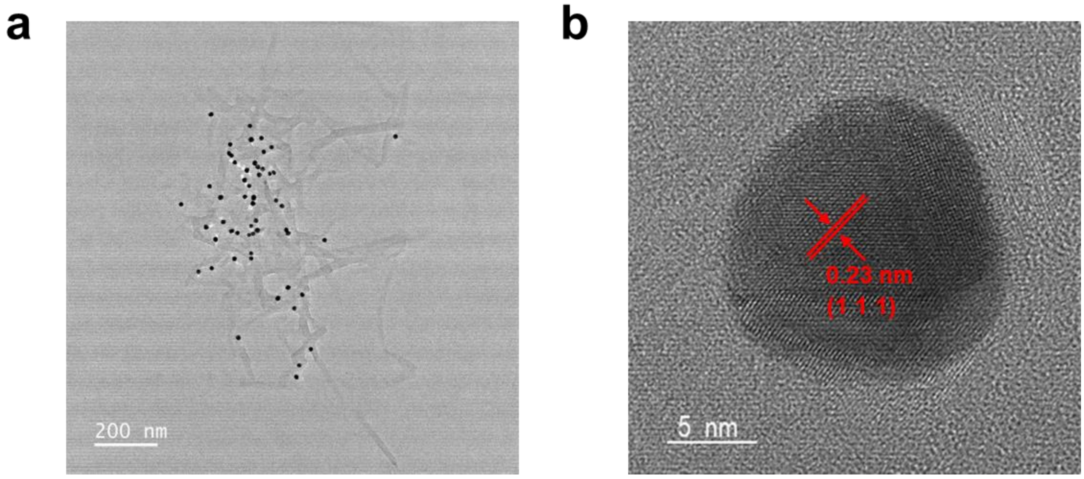

Supplementary Fig. 45. TEM images. (a) HAADF-STEM image of Au NPs/HRP@HOF-100. (b) The high-resolution TEM image of Au NPs. The HAADF-STEM image of Au NPs/HRP@HOF-100 showed the existence of Au nanoparticles onto HOF scaffold (Supplementary Fig. 45a). In addition, the high-resolution TEM image identified the interplanar spacing of 0.23 nm, ascribed to the (111) lattice plane of Au nanoparticles (Supplementary Fig. 45b). These results also demonstrated the successful synthesis of Au NPs/HRP@HOF-100.

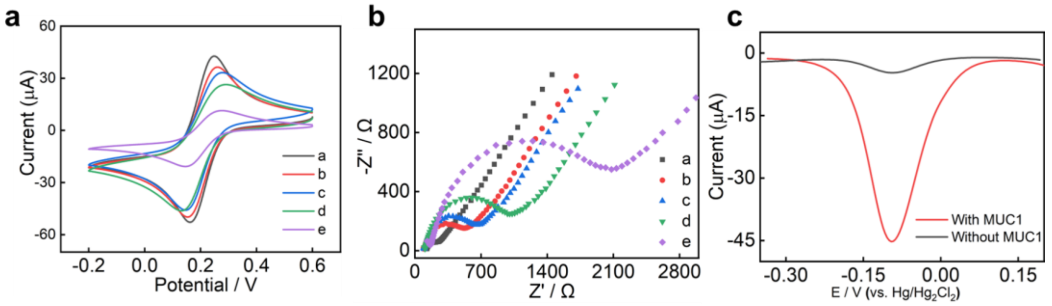

Supplementary Fig. 46. The electrochemical property. CV plots (a) and Nyquist plots (b) of bare GE (curve a), Ab1/GE (curve b), BSA/Ab1/GE (curve c), MUC1/BSA/Ab1/GE (curve d), and Ab2-Au NPs-HRP@HOF-100/MUC1/BSA/Ab1/GE (curve e) in 0.1 M KCl aqueous solution containing 5 mM $[Fe(CN)6]^{3-/4-}$. (c) DPV responses of the proposed biosensor toward 100 ng/mL MUC1 in the PBS solution including 4.5 mM HQ and 3 mM H_2O_2.

Note: To verify the successful fabrication of the proposed biosensor, the cyclic voltammetric (CV) studies were implemented in the presence of $[Fe(CN)_6]^{3-/4-}$ solution (5 mM, acting as the redox probe). As displayed in Supplementary Fig. 46a, a pair of obvious redox peaks were observed on the bare GE (curve a), which proved its own conductivity. After immobilizing Ab1 (curve b), blocking with BSA (curve c), and capturing the MUC1 (curve d), the redox peak currents decreased successively, which was due to the curbing of the electron transfer by non-electroactive proteins. A further decreased redox peak current was acquired when the Ab2-Au-HRP-HOF-100 bioconjugate (curve e) was incubated on the above modified electrode. Moreover, Supplementary Fig. 46b showed the electrochemical impedance spectroscopy (EIS) plots of the sensing method. There was a small semicircle of bare GE (curve a) owing to the low electron-transfer resistance (R_{et}) value of $Fe(CN)_6^{3-/4-}$. The R_{et} values increased successively after stepwise modification of Ab1 (curve b), BSA (curve c), MUC1 (curve d), and Ab2-Au-HRP-HOF-100 (curve e), which was coincided with the result of CV. Additionally, Supplementary Fig. 46c exhibited the DPV curves of the presented electrochemical biosensor with or without target MUC1 in the PBS solution containing HQ and H_2O_2. In the absence of MUC1, the biosensor showed a small current response. However, with the addition of 100 ng/mL target MUC1, the current

significantly increased by 9.6 times due to the H_2O_2-HRP-HQ-mediated signal amplification. These results proved the successful assembly and feasibility of the proposed biosensor.

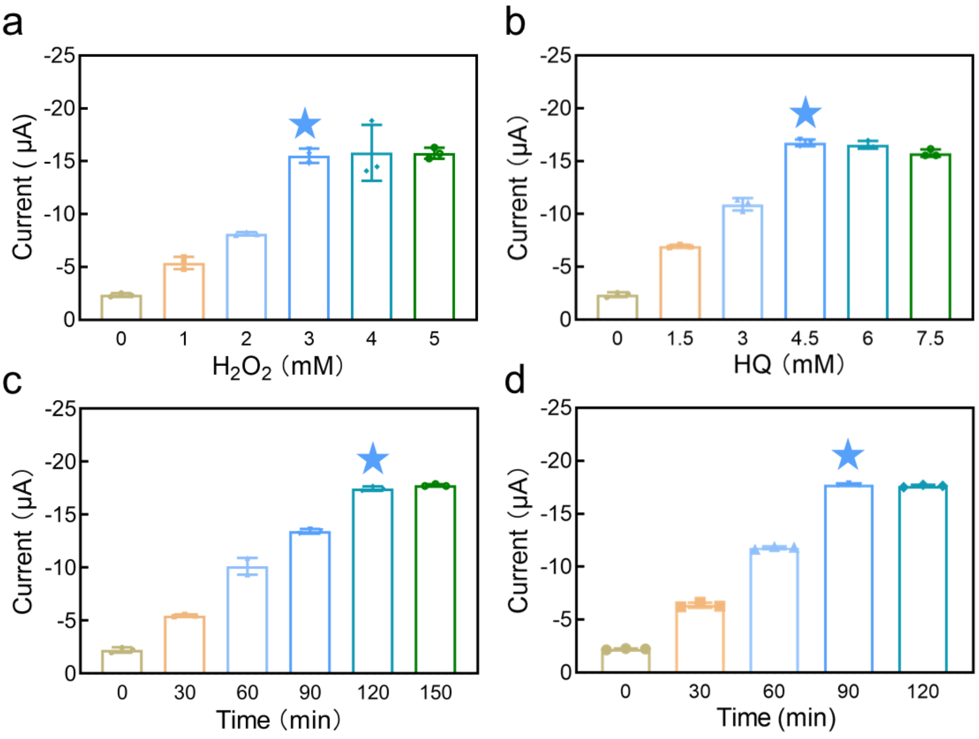

Supplementary Fig. 47. The optimization parameters for electrochemical immunosensor. The effect of the concentrations of H_2O_2 (a), HQ (b), the incubation times of MUC1 (c) and Ab2/Au NPs/ HRP@HOF-100 (d) on the response current of developed biosensor. The data of 3 independent experiments and calculated error bars (SD) are presented in a-d, SD = Standard Deviation (n = 3). Data are presented as mean values +/- SD.

Note: To fulfill the optimal analysis performance of the developed biosensor, several experimental conditions, including the concentrations of HQ and H_2O_2 and the incubation times of MUC1 and Ab2-Au-HRP-HOF-100, were investigated with 10 pg/mL of MUC1 as a model. As illustrated in Supplementary Figs. 47a and b, the maximum current responses were obtained when the optimal concentration of H_2O_2 and HQ were 3.0 and 4.5 mM, respectively. The electrochemical response current increased with the incubation time between MUC1 and BSA/Ab1/GE increased, and then reached

the maximum when the incubation time was 120 min (Supplementary Fig. 47c). Besides, the current response also gradually increased with the enhancement of the binding time between Ab2/Au NPs/HRP@HOF-100 and MUC1/BSA/Ab1/GE, and the maximum current was obtained after 90 min of incubation (Supplementary Fig. 47d). Therefore, 120 min and 90 min were chosen as the optimum combined time for MUC1 and Ab2/Au NPs/HRP@HOF-100, respectively.

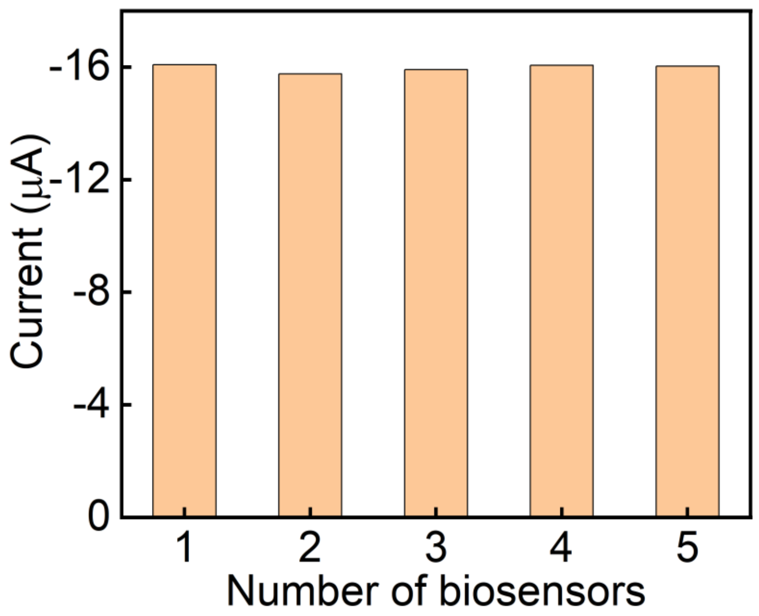

Supplementary Fig. 48. The reproducibility. Reproducibility of the immunosensor in five parallel tests. MUC1 concentration: 10 pg/mL.

Supplementary References

1. Ma, K. et al. Ultrastable Mesoporous Hydrogen-Bonded Organic Framework-Based Fiber Composites toward Mustard Gas Detoxification. *Cell Rep. Phys. Sci.* **1**, 100024 (2020).

2. Wang, C. et al. Dynamic Modulation of Enzyme Activity by Near-Infrared Light. *Angew. Chem. Int. Ed.* **56**, 6767-6772 (2017).

3. Bradford, M. M. A rapid and sensitive method for the quantitation of microgram quantities of protein utilizing the principle of protein-dye binding. *Anal. Biochem.* **72**, 248-254 (1976).

4. Goldring, J. P. D. Measuring Protein Concentration with Absorbance, Lowry, Bradford Coomassie Blue, or the Smith Bicinchoninic Acid Assay Before Electrophoresis. In: *Electrophoretic Separation of Proteins: Methods and Protocols* (ed^(eds Kurien BT, Scofield RH). Springer New York (2019).

5. Meng, X. et al. High-Performance Self-Cascade Pyrite Nanozymes for Apoptosis–Ferroptosis Synergistic Tumor Therapy. *ACS Nano* **15**, 5735-5751 (2021).

6. Ji, X., Song, X., Li, J., Bai, Y., Yang, W. & Peng, X. Size Control of Gold Nanocrystals in Citrate Reduction: The Third Role of Citrate. *J. Am. Chem. Soc.* **129**, 13939-13948 (2007).

7. Chen, A., Ma, S., Zhuo, Y., Chai, Y. & Yuan, R. In Situ Electrochemical Generation of Electrochemiluminescent Silver Naonoclusters on Target-Cycling Synchronized Rolling Circle Amplification Platform for MicroRNA Detection. *Anal. Chem.* **88**, 3203-3210 (2016).

8. Kaminsky, L. S., Miller, V. J. & Davison, A. J. Thermodynamic studies of the opening of the heme crevice of ferricytochrome c. *Biochemistry* **12**, 2215-2221 (1973).

9. Vallée-Bélisle, A. & Michnick, S. W. Visualizing transient protein-folding intermediates by tryptophan-scanning mutagenesis. *Nat. Struct. Mol. Biol.* **19**, 731-736 (2012).
